# Ice-sheet dynamics drive glacial-interglacial shifts in North Atlantic seawater neodymium isotopes

**DOI:** 10.1126/sciadv.aeg5747

**Published:** 2026-08-19

**Authors:** Antao Xu, Kira Just, Alexander M. Piotrowski, Jonathan Stohl, Norbert Frank

**Affiliations:** ^1^ Institute of Environmental Physics, Universität Heidelberg, Heidelberg, Germany.; ^2^ Department of Earth Sciences, University of Cambridge, Cambridge, UK.

## Abstract

Radiogenic neodymium (Nd) isotopic composition (ε_Nd_) of seawater is widely used to reconstruct past ocean circulation, assuming quasi-constant end-member signatures throughout the Quaternary. However, the ε_Nd_ signature of Glacial North Atlantic Intermediate Water deviated markedly from its modern value, challenging this assumption, and the driving mechanisms remain debated. Here, we show that the upper Glacial North Atlantic Intermediate Water was consistently more radiogenic (−7.2) than interglacial values (−13.4) over the past 230,000 years and that pronounced glacial-interglacial ε_Nd_ variability closely followed global ice-volume fluctuations (*r* = 0.80). This variability was primarily reconciled with Icelandic volcanic Nd inputs, modulated by the advance and retreat of the Icelandic Ice Sheet. A forward model reproduces the observed ε_Nd_ evolution and reveals a nonlinear response of Nd export to ice-sheet dynamics, yielding a mean glacial Icelandic Nd input flux of 2.4 × 10^8^ grams per year. Our results highlight the pivotal role of high-latitude ice-sheet systems in regulating North Atlantic seawater chemistry during glacial periods.

## INTRODUCTION

Radiogenic neodymium (Nd) isotopic compositions (expressed as ε_Nd_; see Materials and Methods) are a useful tracer of sediment provenance and the origin and mixing of water masses, serving as proxies for past changes in ocean circulation (*1*–*5*). This is enabled by the quasi-conservative behavior of dissolved Nd in seawater and its shorter oceanic residence time of 300 to 1000 years compared to global seawater mixing time (*6*–*8*). In the modern Atlantic, northern-sourced water (NSW) and southern-sourced water (SSW) masses display distinct ε_Nd_ fingerprinting signatures. In particular, NSWs such as upper and lower North Atlantic Deep Water (NADW) have a ε_Nd_ of ∼−14 to −12 (*9*–*12*) because of weathering input of old, unradiogenic continental rocks around the North Atlantic (*13*), whereas SSWs, including Antarctic Intermediate Water (AAIW) and Antarctic Bottom Water (AABW), are more radiogenic with ε_Nd_ values around −8 reflecting the admixture of Atlantic and Pacific waters (*14*–*16*).

The application of ε_Nd_ as a tracer of past ocean circulation relies on the assumption that the end-member signatures of major water masses (e.g., NADW and AABW) either remained quasi-constant over glacial-interglacial timescales and comparable to their modern values (*17*, *18*) or can be accurately reconstructed for the past interval considered. However, the assumption of quasi-constant end-members is debated for periods of major climatic reorganization such as the Last Glacial Maximum (LGM), when shifts in continental weathering inputs and ocean circulation likely modified the ε_Nd_ signatures of NSW (*19*–*23*). For example, authigenic ε_Nd_ records at ∼40°N indicate that the glacial NADW end-member [referred to as Glacial North Atlantic Intermediate Water (GNAIW)] became more radiogenic, with ε_Nd_ values around −10.0 during the LGM resulting from reduced weathering input of unradiogenic Nd from North American continent (*24*). Waters from the Labrador Sea (ε_Nd_ = −31 to −14) and the Nordic Seas (ε_Nd_ = −15 to −12) during the LGM were too unradiogenic to account for this shift and were thus ruled out as major contributors (*25*, *26*). However, the potential contribution of more radiogenic northeast (NE) Atlantic seawater to the GNAIW end-member was largely overlooked (*27*). Because deep-water formation shifted possibly southeastward to the Iceland Basin with a stronger southeastern recirculation during the LGM (*28*), any seawater ε_Nd_ signature changes in the NE Atlantic could have been incorporated into GNAIW and subsequently exported southward with the overturning circulation, thereby influencing the ε_Nd_ of underlying and downstream water masses (*29*).

**Fig. 1.**
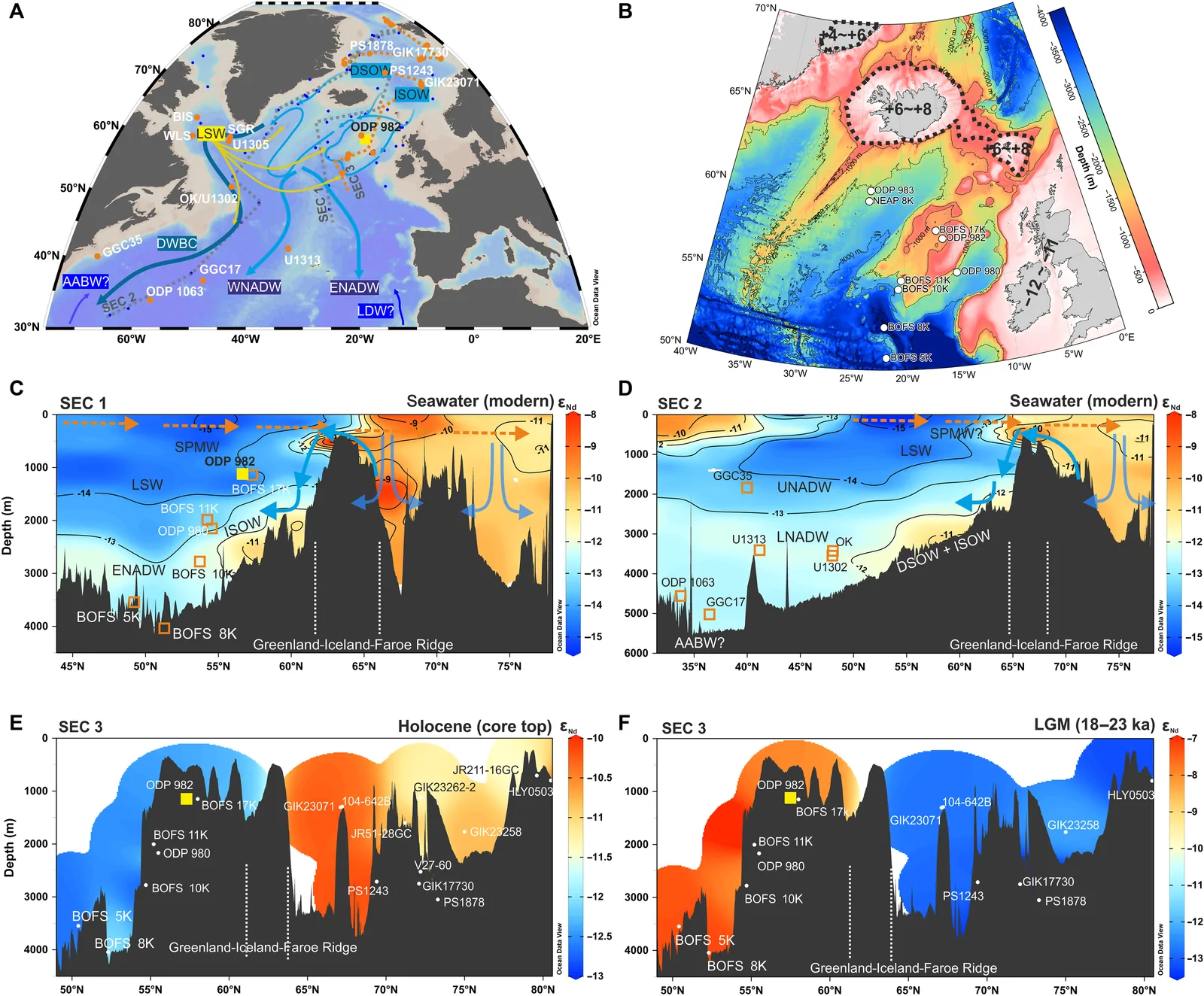
North Atlantic sites and water circulation. (**A**) Locations of seawater (blue dots) and sediment core (orange dots) sites, with section transects (SEC 1, SEC 2, and SEC 3) shown as gray and orange dashed lines. Major intermediate and deep-water masses are indicated by colored arrows, including DSOW (Denmark Strait overflow water), ISOW, LSW, DWBC (Deep Western Boundary Current), WNAWD and ENADW (Western and Eastern North Atlantic Deep Water), AABW, and LDW (Lower Deep Water). (**B**) Bathymetric map of the Iceland Basin showing sampling sites of sediment cores (white dots). Reported ε_Nd_ values for the Greenland-Iceland-Faroe Ridge and the British Isles are indicated. Icelandic bedrock exhibits highly radiogenic ε_Nd_ signatures (+6 to +8) (*49*, *61*). (**C**) Modern seawater ε_Nd_ distribution along SEC 1 from the Nordic Seas to the central North Atlantic. ODP 982 (yellow square) samples are within water masses characterized by unradiogenic ε_Nd_ (−13 to −14). Referencing sediment cores are shown nearby as orange open squares. (**D**) Modern seawater ε_Nd_ distribution along SEC 2 from the Nordic Seas to the western Atlantic, with referencing sediment core sites indicated by orange open squares. (**E**) Sediment-reconstructed modern (core-top) ε_Nd_ along SEC 3 from the Nordic Seas to the NE Atlantic. (**F**) Sediment-reconstructed LGM ε_Nd_ distribution along SEC 3. Nd isotope data are compiled from previous studies (*9*, *12*, *25*–*27*, *50*). The map was created using Ocean Data View (*102*).

To constrain the long-term evolution of the ε_Nd_ signature and seawater chemistry of NE Atlantic thermocline and intermediate waters (upper GNAIW), we present new paired authigenic and residual ε_Nd_ records from ODP Site 982 on the Rockall Plateau (1135 m; Fig. 1). The results show that the upper GNAIW end-member was consistently more radiogenic (∼−7) than modern and interglacial values (−14 to −13) throughout all glacial periods of the past 230,000 years, closely tracking global ice-volume fluctuations. We demonstrate that this pronounced glacial-interglacial variability in seawater ε_Nd_ was primarily regulated by volcanic Nd inputs from Iceland, the dominant radiogenic source in the region, and was strongly modulated by the cyclical advance and retreat of the Icelandic Ice Sheet. These findings reveal a previously overlooked coupling between ice-sheet dynamics, volcanic inputs, and seawater ε_Nd_ in the North Atlantic, with implications for how glacial water-mass end-members are defined and interpreted.

## RESULTS AND DISCUSSION

### Variability and reliability of reconstructed NE Atlantic seawater Nd isotopes

**Fig. 2.**
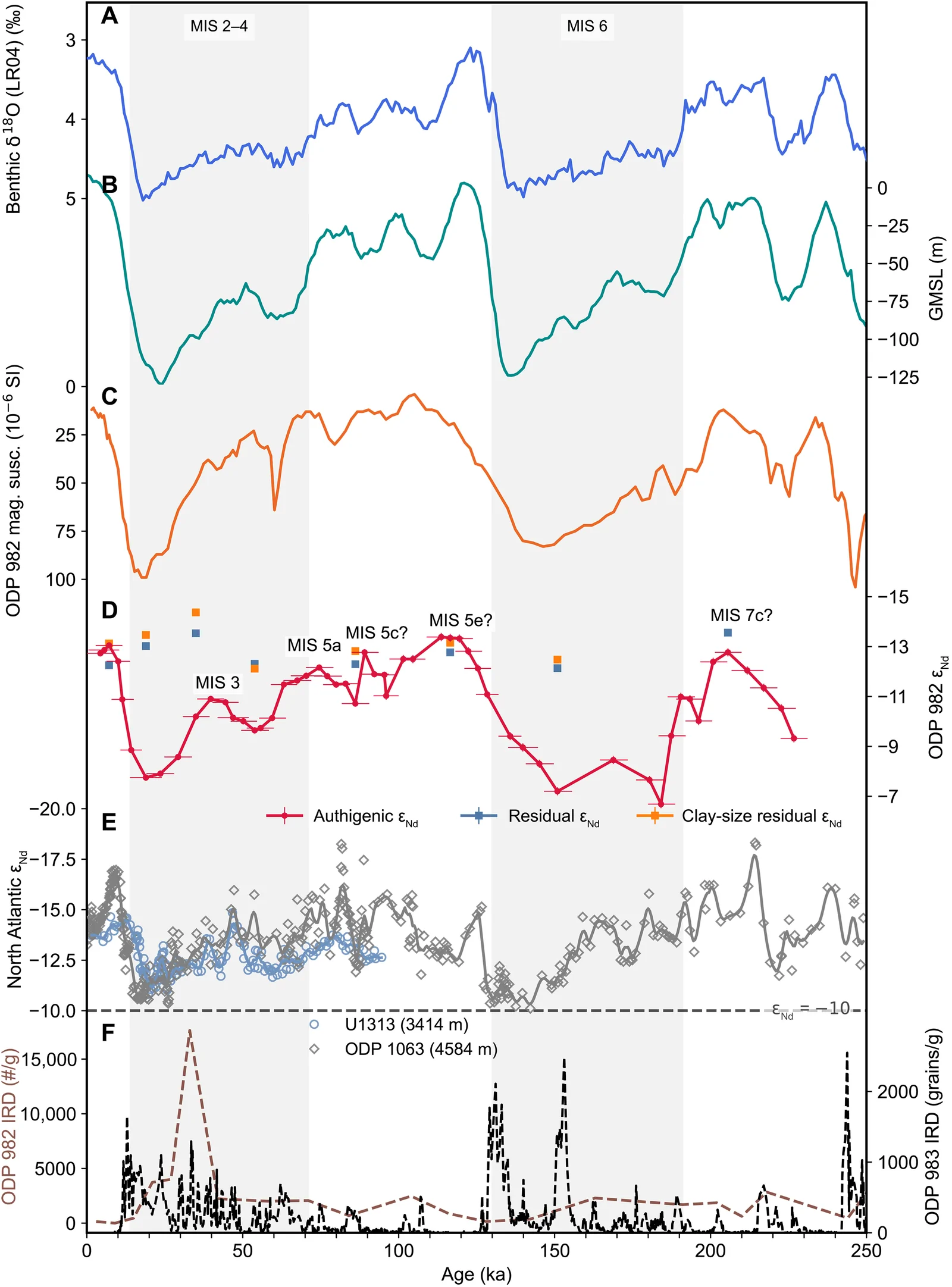
Reconstructed NE Atlantic seawater ε_Nd_ over the past 230,000 years. (**A**) LR04 benthic δ^18^O stack (*91*). (**B**) Reconstructed global mean sea level (GMSL) (*103*). (**C**) ODP 982 magnetic susceptibility record (*104*). (**D**) Authigenic ε_Nd_ record from ODP 982 (red dots) together with residual (blue squares) and clay-size residual fractions (orange squares). Age uncertainty was estimated by the Monte Carlo framework and the details in Materials and Methods and the Supplementary Materials (fig. S2). (**E**) Authigenic ε_Nd_ record from IODP U1313 (*80*, *105*–*108*) and ODP 1063 (*3*, *4*, *109*–*111*), shown as blue circles and gray diamonds, respectively. (**F**) IRD counts from ODP 982 and ODP 983 sediment cores (*112*, *113*).

**Fig. 3.**
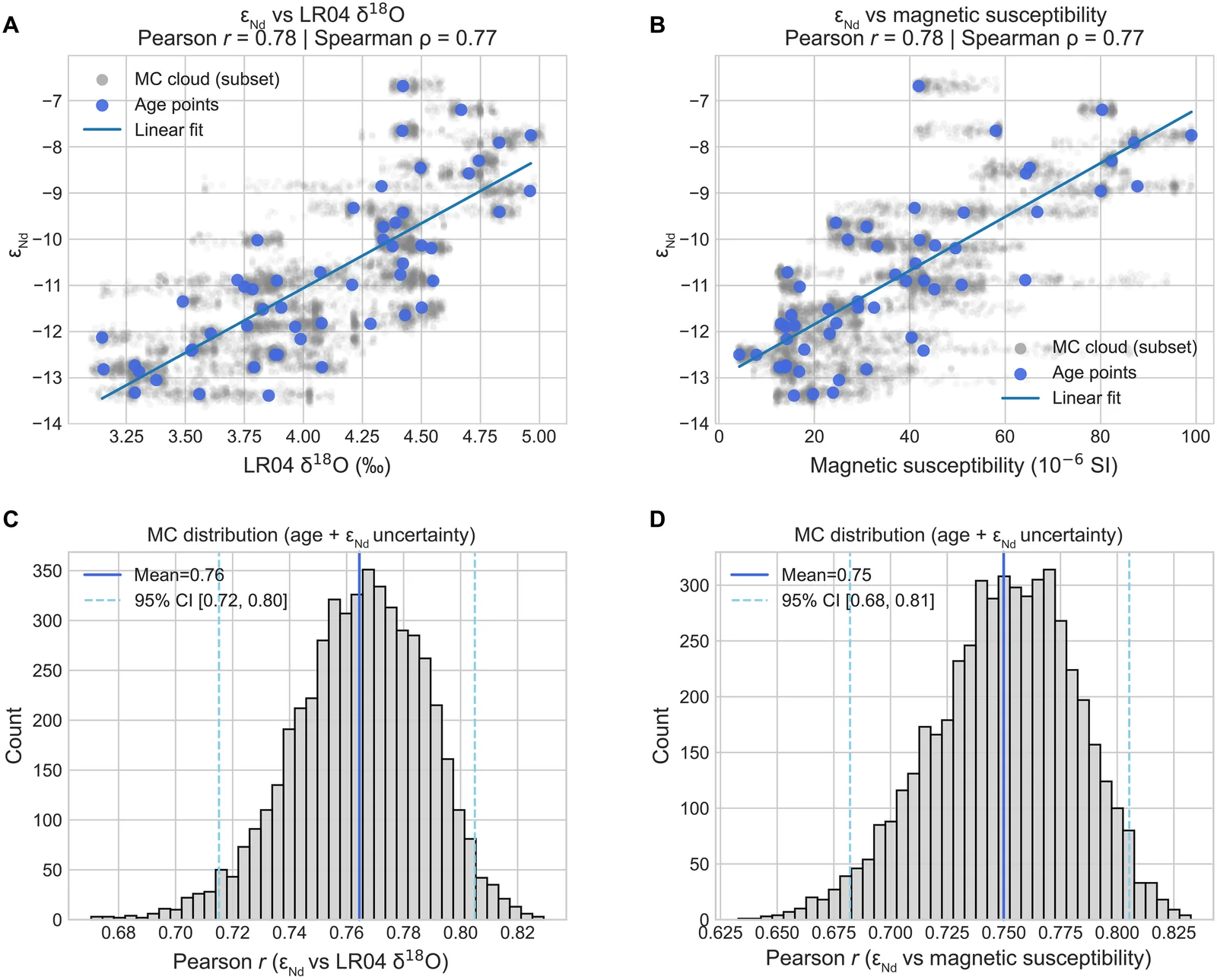
Robustness of ε_Nd_-climate correlations assessed through Monte Carlo (MC) uncertainty propagation. (**A**) ε_Nd_ versus LR04 δ^18^O (blue points). The gray cloud represents a subsample of MC realizations (joint age and ε_Nd_ uncertainty). The solid line shows the least-squares fit to the data. (**B**) Same as (A) but for ε_Nd_ versus magnetic susceptibility. (**C** and **D**) Monte Carlo distributions of Pearson’s *r* for ε_Nd_-LR04 δ^18^O (C) and ε_Nd_-magnetic susceptibility (D), showing the mean values (solid line) and 95% CI (dashed).

The downcore record of authigenic ε_Nd_ from ODP Site 982 reveals pronounced glacial-interglacial variability over the past ∼230,000 years (Fig. 2D). Values of ε_Nd_ range from highly unradiogenic signatures of −12.9 ± 0.2 (*n* = 3, ±1 SD) in Holocene, −13.4 ± 0.03 (*n* = 3, ±1 SD) at Marine isotope stage (MIS) 5e, and −12.8 at MIS 7c to the most radiogenic (positive) signatures of −7.2 ± 0.5 (*n* = 3, ±1 SD) during the MIS 2 and MIS 6 glacial maxima. Other ε_Nd_ values fall between these extremes. More unradiogenic (negative) ε_Nd_ values during interglacial and interstadial periods are similar to values of −13.5 to −14.0 in present-day Labrador Sea–derived water masses, likely Labrador Seawater (LSW) and/or Subpolar Mode Water at comparable depths near Site 982 (*12*). By contrast, ε_Nd_ values shift markedly toward more positive during the LGM (ε_Nd_ = −7.7 to −7.9) and MIS 6 glacial maximum (ε_Nd_ = −6.7 to −7.7), coinciding with the periods of maximum global ice volume. The glacial-interglacial ε_Nd_ variations closely track the LR04 benthic δ^18^O stack, the reconstructed global mean sea level, and the ODP 982 magnetic susceptibility record (Figs. 2 and 3). More positive ε_Nd_ values coincide with glacial conditions (high δ^18^O and magnetic susceptibility), whereas more negative values occur during interglacials. These relationships are strong and robust (Pearson *r* = 0.80 for δ^18^O and *r* = 0.82 for magnetic susceptibility) and remain essentially unchanged when analytical and age uncertainties are propagated using Monte Carlo (MC) ensembles (*r* = 0.78 ± 0.05 for both proxies). This coupling persists even on stadial and interstadial timescales, thus suggesting that ε_Nd_ variability was paced by processes linked to the cyclic growth and decay of global ice volume and climate conditions.

To verify that this variability truly reflects past seawater composition rather than diagenetic overprinting in sediment (*30*), we further assess the reliability of the reconstructed authigenic ε_Nd_ record. The uppermost samples (top 25 to 40 cm) of this core, as well as the samples from MIS 5e and MIS 7c, closely match modern ε_Nd_ values of seawater in the NE Atlantic (offsets <1 ε_Nd_ unit) (*12*) and the previously reconstructed Holocene values (*27*, *31*, *32*), demonstrating the reliability of the reconstructed seawater ε_Nd_ for the warm climate periods. The residual and clay-size residual ε_Nd_ values of ODP 982 are less positive compared to the Rockall Trough slope sediments of −10.4 ± 1.4 (*31*) but consistent through glacial-interglacial cycles of −12.7 ± 0.6 (*n* = 8, ±1 SD) for residue and −13.1 ± 0.7 (*n* = 7, ±1 SD) for clay-size residue. This consistency indicates an absence of substantial reactive radiogenic Nd sources in sediments, ruling out diagenetic modification as the cause of the more positive authigenic ε_Nd_ signals (*27*, *33*, *34*). Thus, the positive shifts in ε_Nd_ of the authigenic phase during glacial periods primarily reflect changes in seawater isotopic composition rather than sedimentary overprinting. Although glacial sediments may contain higher proportions of ice-rafted debris (IRD) or volcanic particles, and sediment-porewater-bottom water Nd exchange could contribute to this pattern, these processes alone are unlikely to account for the basin-scale, depth-integrated, and climate-paced ε_Nd_ shifts observed (*27*). The seawater origin of the Nd isotope signal is further supported by rare earth element (REE) patterns and element ratios of the leachates, which show a middle-REE bulge, negative cerium anomaly, and low aluminum-to-Nd ratios (*5*–*25*), well below the threshold of 100 (fig. S1) (*35*, *36*). These characteristics confirm minimal detrital contamination and a dominantly authigenic phase that faithfully records ambient seawater isotopic composition.

### Challenges to a water mass mixing explanation for ε_Nd_ variability

Under the assumption of fixed end-member compositions commonly used in paleoceanographic reconstruction, the highly radiogenic values (−7.2 ± 0.5) observed in the upper GNAIW during glacials cannot be explained by any known water mass in either the modern or glacial North Atlantic (Table 1). LSW is far too unradiogenic (ε_Nd_ = −14) to contribute to the positive shift (*9*, *37*, *38*). To evaluate whether other radiogenic water masses could account for the glacial ε_Nd_ signatures at Site 982, we consider potential contributions from the south (e.g., AAIW), the Mediterranean [Mediterranean Outflow Water (MOW)], and the Nordic Seas [Iceland-Scotland Overflow Water (ISOW)] (Table 1). Modern AAIW has a ε_Nd_ value of ∼−8 (*14*, *15*), but its signal becomes strongly diluted northward and its influence was further diminished in the tropical Atlantic during the LGM (*39*–*41*), making its penetration into the NE Atlantic highly improbable. Similarly, MOW, characterized by ε_Nd_ values typically ranging from −9 to −10 in modern and glacial conditions (*42*–*47*), would have undergone strong dilution along its northward pathway to the Iceland. Consequently, it is highly unlikely that SSWs (i.e., AAIW and MOW) are responsible for the findings at ODP 982.

**Table 1.** Neodymium isotopic signatures (ε_Nd_) of the different end-members.

	Seawater/authigenic ε_Nd_		Detrital/residual ε_Nd_	
	Modern/Holocene (<5 ka)	LGM (18–23 ka)	Modern/Holocene (<5 ka)	LGM (18–23 ka)
Labrador Seawater	−13.7 ± 0.9 (*9*), −13.9 ± 0.4 (*37*)	—	—	—
Antarctic Intermediate Water	−8.1 ± 0.2 (*14*)	—	—	—
Mediterranean Outflow Water	−9.8 ± 0.6 (*115*), −9.4 ± 0.5 (*43*)	−9.1 ∼ −8.9 (*44*)	—	—
Iceland-Scotland Overflow Water	−8.2 ± 0.6 (*50*)	—	—	—
Upper and lower North Atlantic Deep Water	−13.2 ± 1.0, −12.4 ± 0.4 (*9*)	—	—	—
Labrador Sea (reconstruction)	−15.8 ∼ −14.0 (*56*, *57*)	−30.6 ∼ −13.7 (*56*, *57*)	−19.3 ∼ −18.7 (*57*)	−28.8 ∼ −20.0 (*57*)
Norwegian and Greenland Seas (reconstruction)	−10.8 ∼ −9.7 (*25*, *26*)	−14.7 ∼ −12.1 (*25*, *26*)	−12.7 ∼ −12.6 (*25*)	−14.7 ∼ −14.0 (*25*)
Mid-depth North Atlantic (reconstruction)	−14.3 ∼ −14.1 (*24*)	−10.2 ∼ −9.6 (*24*)	−12.6 (*24*)	−10.5 ∼ −9.3 (*24*)
Deep North Atlantic (reconstruction)	−15.9 ∼ −13.3 (*3*, *106*, *110*)	−12.6 ∼ −10.6 (*3*, *106*, *107*, *110*, *111*)	—	—
NE Atlantic: ODP Site 982 (1135 m, this study)	−13.0 ∼ −12.7	−7.7	−12.3	−13.0
NE Atlantic: BOFS (1000–4000 m)	−13.7 ∼ −11.1 (*27*)	−9.6 ∼ −4.2 (*27*)	−12.7 ∼ −10.7 (6–7 ka) (*27*)	−12.6 ∼ −11.4 (*27*)
Iceland input	—	—	+4 ∼ −8 (*27*, *49*, *61*, *63*)	+4 ∼ −8 (*27*, *49*, *61*, *63*)

**Fig. 4.**
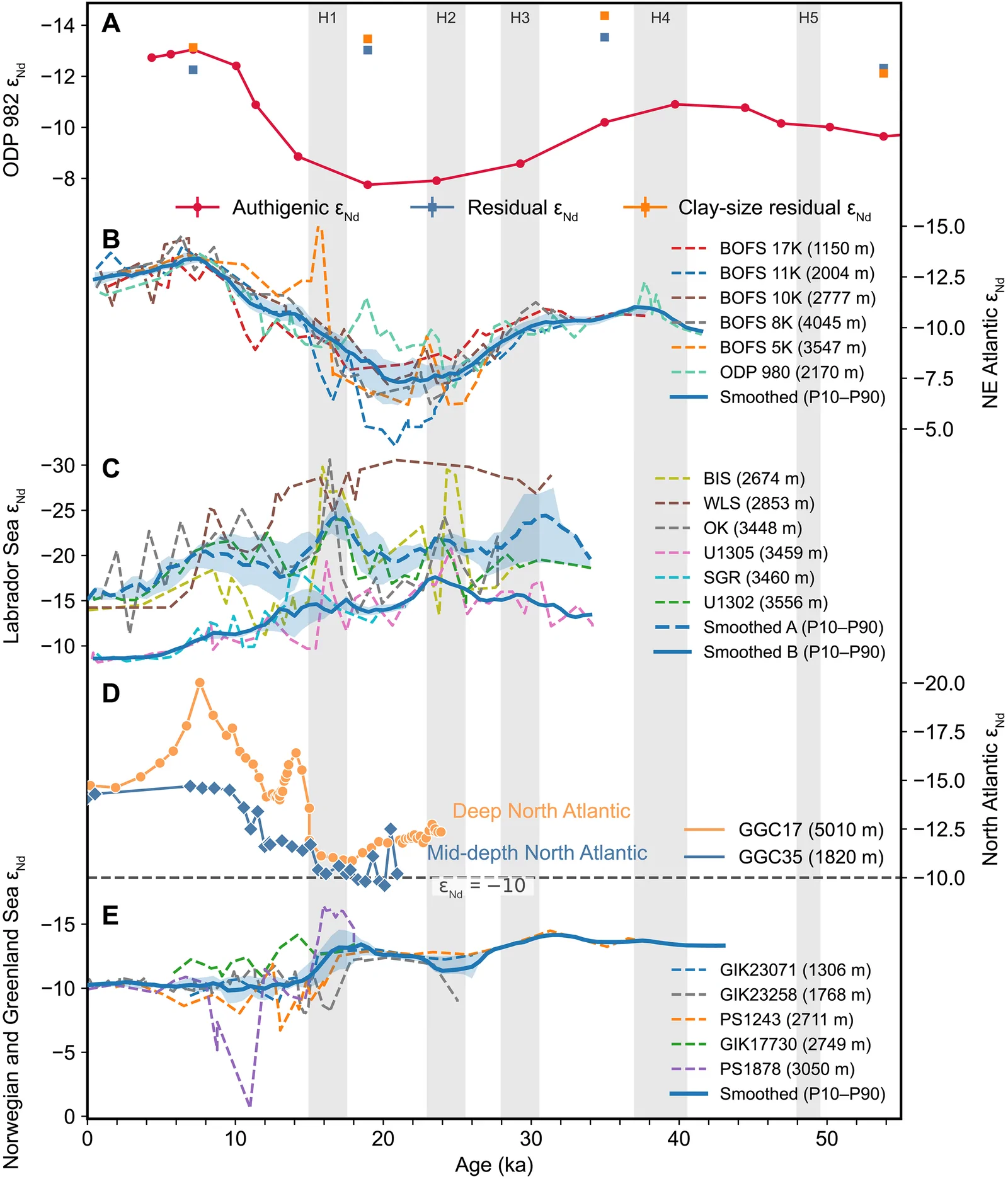
Comparison of NE Atlantic ε_Nd_ with other regional sediment cores over the past 50,000 years. (**A**) Authigenic ε_Nd_ record from ODP Site 982 (red dots) and corresponding residual (blue squares) and clay-size residual fractions (orange squares). (**B**) Authigenic ε_Nd_ records from BOFS sediment cores (*27*) and ODP Site 980 (*55*). All core data are smoothed (blue line). (**C**) Authigenic ε_Nd_ records from the Labrador Sea (*56*, *57*). Data from Sites U1305 and SGR were smoothed by the blue line, while other core data were smoothed by the dashed line. (**D**) Authigenic ε_Nd_ records from mid-depth and deep North Atlantic sites (*24*, *106*). (**E**) Authigenic ε_Nd_ records from the Norwegian and Greenland Seas (*25*, *26*). Blue lines with shaded bands show smoothed ε_Nd_; the shaded area denotes the P10-to-P90 range (10th to 90th percentiles) from bootstrap + Monte Carlo resampling based on ε_Nd_ measurement uncertainties.

Another candidate is ISOW, with present-day ε_Nd_ values of −8.2 ± 0.6 for pure ISOW, reflecting boundary exchange with radiogenic basaltic bedrock (ε_Nd_ = +6 to +8) of the Greenland-Iceland-Faroe Ridge (*48*–*50*). However, even a 100% ISOW contribution cannot reproduce the most positive values at Site 982 (−6.7) or nearby BOFS cores (−4.2) (*27*). Hence, ISOW with its modern isotopic composition cannot account for the observed glacial ε_Nd_ shifts. Even if ISOW possibly acquired a more radiogenic signature in glacial periods via prolonged seawater-margin/boundary interaction (*51*, *52*), because of diminished formation and slower overflow export during glacial periods (*53*), subsequent mixing would strongly dilute its signature. As observed today, ISOW typically attains ε_Nd_ values of −10.5 to −11.5 in the NE Atlantic after entrainment with overlying Atlantic waters and LSW (Fig. 1C) (*12*, *50*). Given this inevitable dilution forced by the Bernoulli effect, it is mechanically improbable that a weaker overflow could dominate radiogenic ε_Nd_ signatures throughout the 1000- to 4000-m water column (Fig. 4B) (*27*). Moreover, explaining the entire water column ε_Nd_ shifts with single ISOW would require vigorous vertical mixing, which is contradicted by strong stratification during the LGM indicated by biological proxies (*54*). Therefore, the observed ε_Nd_ variability at Site 982 cannot be explained solely by varying proportions of known water masses (Table 1), requiring the addition of an external radiogenic Nd source.

### Ice sheet–modulated volcanic erosion inputs as drivers of seawater ε_Nd_ evolution

The external radiogenic Nd source previously proposed for nearby BOFS cores is volcanic IRD from Iceland (Fig. 4) (*27*). Several observations, however, indicate that IRD pulses alone cannot explain the ε_Nd_ structure at ODP Site 982: (i) The most positive ε_Nd_ shifts in the BOFS records occur during the LGM rather than aligning with individual Heinrich IRD events. If IRD delivery were the primary control, peak ε_Nd_ should coincide with discrete IRD pulses and exhibit rapid, high-amplitude fluctuations. Instead, at Site 982, we observe no clear relationship between IRD counts in the Iceland Basin (ODP 982/983) and authigenic ε_Nd_ (Fig. 2F). This is consistent with three constraints: (i) IRD can only transiently “relabel” seawater ε_Nd_ because debris sink rapidly, and any imprint on authigenic phases is expected to be confined to discrete IRD-rich layers (*27*, *55*–*57*); (ii) radiogenic Nd released from Icelandic basaltic IRD (ε_Nd_ > +4) and unradiogenic Nd associated with Laurentide-derived IRD (ε_Nd_ < −20) can partly offset each other on a regional scale, damping the net seawater signal; and (iii) low sedimentation rates at ODP Site 982 [2.1 ± 1.0 cm per thousand years (ka), 1 SD, *n* = 54] combined with 1-cm-thick sample slices imply that each authigenic Nd sample integrates seawater signals over several hundred years, potentially exceeding ∼1000 years during glacial maxima, thereby smoothing short-lived IRD-driven variability. (2) Neither BOFS nor Site 982 shows a robust relationship between volcanic IRD abundance and authigenic ε_Nd_ on glacial-interglacial timescales, implying that the mere presence of volcanic debris is not the dominant control on the long-term seawater signal. (3) Instead, ODP 982 ε_Nd_ correlates strongly with global ice volume (Fig. 3), suggesting that ε_Nd_ responds to processes whose intensity scales with ice-sheet growth and decay. We do not exclude local, short-lived IRD impacts: In BOFS 5K, ODP 980, and Labrador Sea records, unradiogenic ε_Nd_ excursions coincide with Heinrich events (Fig. 4, B and C), likely reflecting strong Laurentide or British-Irish ice-sheet inputs when these IRD fluxes overwhelm Icelandic contributions. However, these signals are spatially restricted to the IRD belt and are too brief to explain the persistent, climate-paced ε_Nd_ structure at Site 982. Collectively, the evidence points to a more continuous radiogenic Nd supply modulated by ice-sheet evolution and climate-driven changes in reactive Nd supply (*58*). Volcanic ash may contribute intermittently; however, mean eruption rates increased after deglaciation at ∼12 ka because of ice-sheet unloading (*59*), which does not necessarily translate into stronger radiogenic inputs to the NE Atlantic, because glacial erosion, comminution, and sediment transport pathways were reduced relative to full-glacial conditions. A small positive ε_Nd_ excursion observed at BOFS 17K may reflect a localized response to enhanced volcanic activity during this interval. In contrast, neither Site 982 nor the other BOFS records show a distinct basin-wide radiogenic peak around ∼12 ka, suggesting that such signals were either attenuated by low sedimentation rates and limited sampling resolution or restricted to spatially limited regions (*60*).

In the North Atlantic, Iceland represents the most plausible and dominant source of radiogenic lithogenic material, with ε_Nd_ values of +4 to +8 reported for Icelandic bedrock, volcanic debris, and detrital sediments (*49*, *61*–*63*). Modern observations demonstrate that basaltic inputs can strongly modify seawater ε_Nd_: Icelandic particles can shift Atlantic-derived waters from ∼− 15 to −16 toward −6 to −3 accompanied by a >50% increase in dissolved Nd concentrations, although this influence is largely confined to nearshore regions by coastal circulation (*60*). In the Denmark Strait, intense seawater-margin exchange yields seawater ε_Nd_ of ∼−4 to −2 at 0 to 300 m above the Iceland shelf (*64*), illustrating that when exchange and circulation pathways are favorable, Icelandic inputs can substantially reshape regional seawater ε_Nd_ distribution. During the LGM, the Icelandic Ice Sheet likely amplified this effect by enhancing the production and export of reactive basaltic material, while the lowered sea level expanded shelf exposure and promoted more efficient seawater-margin exchange. Reconstructions suggest a large maximum ice-sheet extent with relatively shallow geometry and widespread basal sliding (*65*), conditions that promote efficient subglacial erosion, high sediment yields, and rapid export via ice streams. Geological and seismic evidence further indicates maximum sediment delivery when the ice margin advanced to the shelf-slope break, associated with intense erosion and the deposition of thick glacigenic wedges (*66*). Mineralogical and magnetic constraints also support enhanced Iceland-sourced sediment contributions during the LGM, delivered to the Denmark Strait via ice-stream discharge and iceberg calving (*67*). Consistent with this mechanism, Site 982 ε_Nd_ covaries with available reconstructions of Icelandic ice-sheet evolution over the past ∼120,000 years (fig. S3) (*68*).

The transfer of this Iceland-sourced radiogenic Nd to NE Atlantic seawater likely involved multiple, synergistic pathways. The dominant mechanism was partial dissolution of subglacially derived reactive particles in the water column. Subglacial comminution produces large amounts of such material, particularly fine-grained glacial flour with high-specific-surface-area and reactive coatings and elevated trace-element leachability (*20*, *69*). Although ε_Nd_ data specific to this fine fraction remain limited, Nd isotopes show no marked grain-size dependence within a given provenance, so glacial flour is expected to share the same radiogenic signature as coarser detrital sediments derived from the same basaltic source (+4 to +8) (*49*, *61*–*63*). Because Icelandic basalt is Nd-rich and highly radiogenic, relatively modest fluxes of such reactive material can disproportionately shift seawater ε_Nd_ (*61*, *70*). Once exported to the ocean, these particles undergo partial dissolution during settling, imprinting a radiogenic signature on ambient waters at multiple depth levels on timescales of days to months (*71*). Benthic flux and boundary exchange along the Icelandic margin (*30*, *34*, *50*) further contributed by labeling bottom waters in contact with a reactive basaltic substrate, although these bottom-up processes alone cannot account for the tight correlation between ε_Nd_ and ice-volume proxies or the disproportionately radiogenic values during rapid glaciation intervals (MIS 6). At Site 982, residual ε_Nd_ remains comparatively stable through glacial-interglacial cycles (−12.7 ± 0.6 for bulk residue; −13.1 ± 0.7 for clay-size residue), indicating limited changes in local detrital provenance. The pronounced decoupling between stable detrital ε_Nd_ and strongly varying seawater ε_Nd_ suggests that the seawater signal is not governed by local detrital inputs at the site. Instead, the radiogenic shift is more consistent with upstream modification of seawater ε_Nd_ closer to Iceland, with the modified water masses subsequently advected to the Rockall Plateau. The near-uniform glacial ε_Nd_ shift observed across the ∼4000-m water column in the NE Atlantic further points to a critical amplifying role of deep-water formation, whereby surface and intermediate waters that had acquired a radiogenic signature were subsequently downwelled as deep-water formation shifted southward during the LGM (*28*), efficiently transmitting the signal throughout the water column. Particle-driven reversible scavenging may have further assisted in redistributing the radiogenic signal to deeper levels (*72*).

**Fig. 5.**
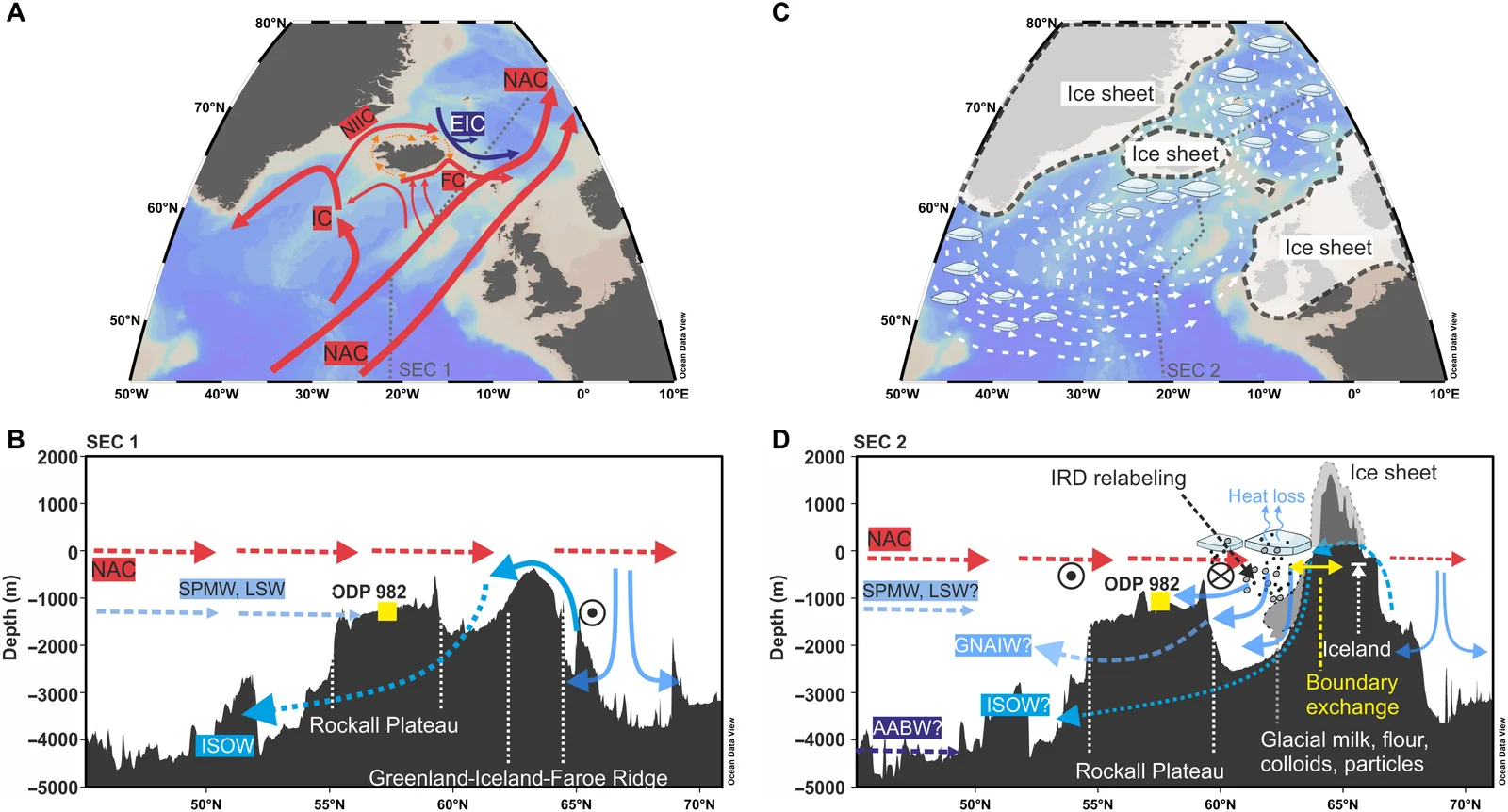
Conceptual diagram illustrating Icelandic control on NE Atlantic ε_Nd_ evolution. Schematic representation of surface and intermediate currents and Nd source dynamics under (**A** and **B**) interglacial and (**C** and **D**) glacial maximum conditions. Surface and intermediate currents include NAC, IC (Irminger Current), FC (Faroe Current), NICC (North Icelandic Irminger Current), and EIC (East Icelandic Current). In (C), white arrows show streamlines of sea-ice drift (*114*). During interglacials, limited Icelandic erosion and weak volcanic material input, together with vigorous surface inflow from the Atlantic to the Nordic Seas, result in less radiogenic ε_Nd_ in the NE Atlantic. During glacials, intensified glacial erosion, subglacial supply, IRD relabeling, and boundary exchange increase Icelandic Nd export, yielding more radiogenic seawater ε_Nd_. Simultaneously, reduced northward transport of the NAC and southward shifts in deep-water formation enhance the influence of Iceland-derived Nd. This framework links high-latitude cryospheric and volcanic fluxes to basin-scale Nd isotope evolution across glacial-interglacial cycles. The figure was created using Ocean Data View (*102*).

We synthesize these processes in Fig. 5. In the modern ocean, the North Atlantic Current (NAC) bifurcates around Iceland, routing surface waters eastward toward the Iceland-Faroe Ridge or westward toward the Irminger Sea, with part of the flow continuing into the Nordic Seas (Fig. 5A). This circulation geometry helps confine Iceland input to nearshore regions and maintain a basin-scale ε_Nd_ gradient, with less radiogenic values (−14 to −13) south of the Ridge and more radiogenic values (−11 to −9) in the Nordic Seas, sustained by basaltic margin exchange and water-mass mixing (Fig. 1, C to E). During the LGM, this gradient was reversed (Fig. 1F), indicating a redistribution (“see-saw”) of Iceland’s geochemical influence between the Iceland Basin and the Nordic Seas. We attribute this reversal to the coupling of ice-sheet dynamics and glacial circulation: (i) Expanded sea ice reduced northward NAC transport (at least seasonally) (*73*), favoring the retention of subglacially derived material and meltwater inputs within the Iceland Basin rather than export to the Nordic Seas; (ii) the lower sea level exposed a broader Icelandic shelf, enlarging the source area for both particle delivery and seawater-basalt contact; and (iii) deep-water formation shifted southward toward the Iceland Basin (*74*, *75*), providing the convective pathway that transferred the radiogenic signal to depth, as discussed above. A relatively weakened Atlantic overturning circulation likely prolonged water-mass residence times (*28*, *76*), enhancing seawater ε_Nd_ relabeling. In parallel, weathering from Scandinavian and Barents ice sheets supplied unradiogenic Nd to the Nordic Seas (*25*), reinforcing the basin-scale contrast.

**Fig. 6.**
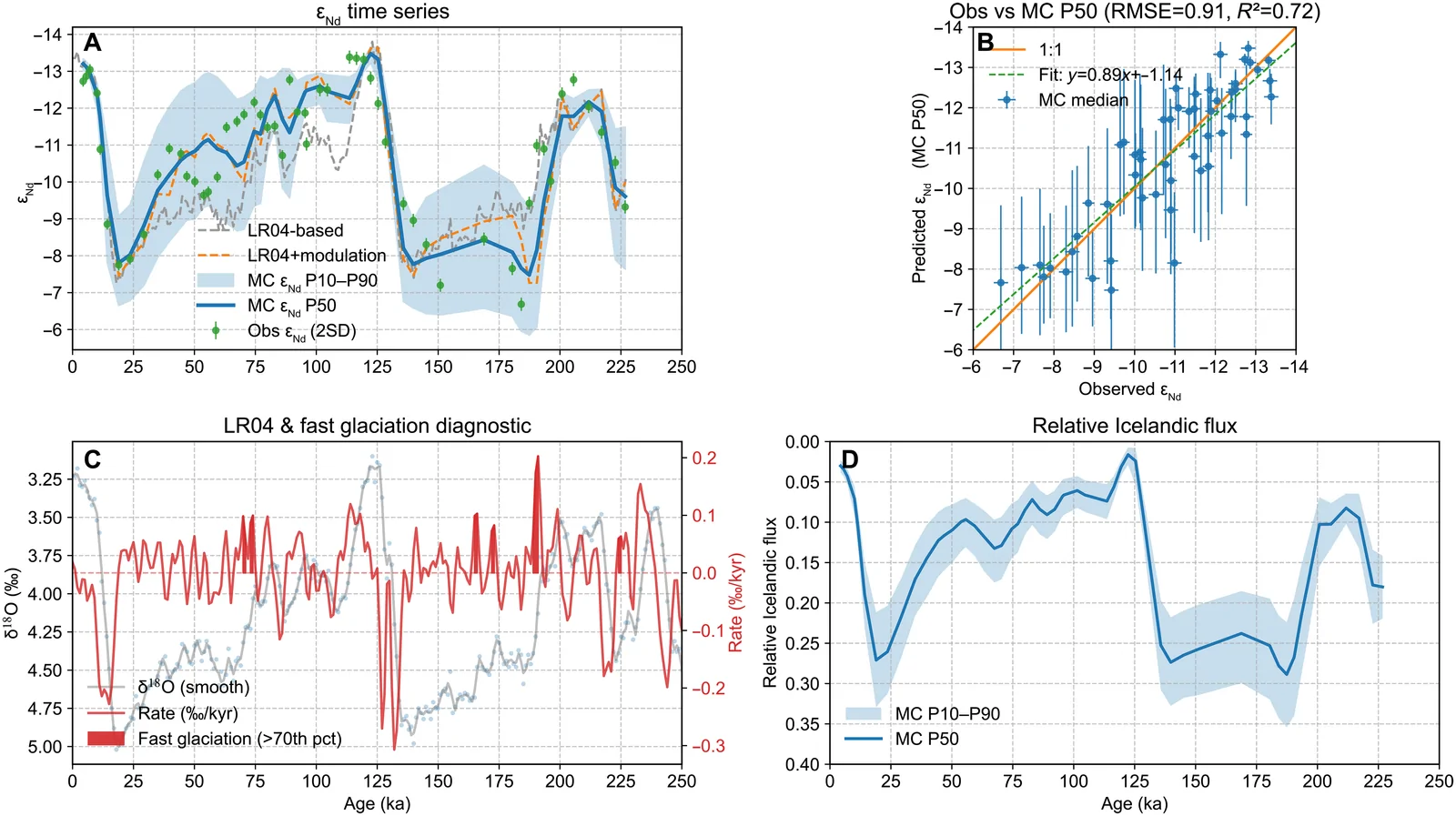
NE Atlantic ε_Nd_ variability and relative Icelandic Nd flux: Baseline model and Monte Carlo ensemble. (**A**) ε_Nd_ time series showing the LR04-based baseline (gray dashed line), the deterministic LR04 with dual modulation (orange dashed line), the MC percentile band P10 to P90 with median P50 (blue), and observations with 2 SD uncertainties (green points). (**B**) Observed ε_Nd_ versus MC median prediction with asymmetric MC uncertainty (P10 to P90) and 2 SD analytical errors; the 1:1 line and an ordinary-least-squares fit are shown. (**C**) LR04 diagnostics: smoothed δ^18^O (gray line) and expansion rate referred from δ^18^O change rates (red line) with “fast glaciation” highlighted where positive rates exceed the 70th percentile. kyr, thousand years. (**D**) Relative Icelandic Nd flux from the MC ensemble (P10 to P90 and P50). The model quantifies the relative Icelandic radiogenic Nd contribution required to reproduce the observed ε_Nd_ variability and indicates how Icelandic ice-sheet dynamics modulate NE Atlantic seawater chemistry. Further details are provided in Materials and Methods and the Supplementary Materials (tables S1 to S3).

To test whether the observed glacial-interglacial ε_Nd_ variability in the NE Atlantic can be explained simply as a linear response to Iceland Ice Sheet evolution, we developed a forward mixing model with MC uncertainty propagation. The model treats seawater ε_Nd_ as binary mixing between an NE Atlantic seawater background (ε_Nd_ = −13.8 for interglacial periods; varied from −16.0 to −10.0 during glacials to account for potential shifts in the ε_Nd_ value of North Atlantic seawater) and an Icelandic volcanic end-member (ε_Nd_ = +4 to +8), with the Icelandic contribution scaled by a glaciality index derived from the LR04 benthic δ^18^O stack. Rather than explicitly resolving water-mass fluxes or circulation geometry, which remains poorly constrained for glacial periods, the model parameterizes the net Icelandic influence as an effective mixing fraction that scales with ice volume, allowing us to test whether ice volume–paced Icelandic inputs alone are sufficient to reproduce the observed ε_Nd_ evolution and to quantify the relative Icelandic contribution and its uncertainty. Because a continuous, high-resolution reconstruction of Icelandic Ice Sheet variability is not available for the full interval and as the growth and retreat of the Icelandic Ice Sheet were broadly synchronous with global ice-volume fluctuations (*66*, *67*, *77*), we use the LR04 benthic δ^18^O stack as a first-order glaciality index, acknowledging that LR04 integrates global ice volume and deep-ocean temperature and may not capture the full regional dynamics of the Icelandic Ice Sheet. After normalization and scaling to the LGM ε_Nd_ target (−7.7; gray dashed line in Fig. 6A), the baseline captures the overall amplitude but underestimates the MIS 6 maximum and remains systematically too positive during MIS 5–3 (*R*^2^ = 0.53; fig. S4). These mismatches suggest that the Icelandic imprint is not strictly linear with global glaciality, likely reflecting the combined effects of regional ice dynamics, circulation geometry (e.g., NAC strength and pathway), and age uncertainty. We therefore introduce a physically motivated nonlinear modulation that reduces the Icelandic contribution during MIS 5–3 and enhances it during MIS 6, with additional amplification during intervals of rapid glaciation inferred from LR04 δ^18^O change rates (Fig. 6C). We propagate age, analytical, and end-member uncertainties using a 2000-member MC ensemble. The model reproduces the observed ε_Nd_ evolution [RMSE (root-mean-square error) = 0.91; *R*^2^ = 0.72] and captures the full range within the P10-to-P90 envelope (Fig. 6). Notably, the ensemble indicates that rapid ice expansion superimposed on cold background conditions leads to a disproportionate increase in the Icelandic contribution. This behavior coincides with the most positive ε_Nd_ values observed during MIS 6 and occurs synchronously with the interval of maximum and rapidly increasing magnetic susceptibility at ODP Site 983 (fig. S5), located upstream and closer to Iceland than ODP 982. The temporal alignment between enhanced lithogenic input at ODP 983 and peak ε_Nd_ at ODP 982 strongly supports our interpretation that fast glaciation promotes intensified erosion, comminution, and export of Icelandic material, resulting in a nonlinear and pulse-like Icelandic imprint on downstream seawater ε_Nd_. Conversely, the weaker-than-expected imprint during MIS 5–3 is consistent with reduced lithogenic export and/or circulation conditions that limit the transfer of Icelandic inputs into the NE Atlantic. The nonlinear behavior could arise from a shelf-edge control, with maximal comminution and export occurring when the Icelandic Ice Sheet reached the continental shelf, whereas rapid retreat onto land would sharply reduce the downstream ε_Nd_ impact. Overall, the results support the view that ice sheet–modulated Icelandic inputs, interacting with glacial circulation, are sufficient to drive the coherent, ice volume–paced ε_Nd_ signal observed at Site 982. Consistent relationships between seawater ε_Nd_ and global ice volume have also been documented on much longer timescales, extending back to the past 1.7 million years, suggesting that ice sheet–regulated weathering and circulation processes exert a persistent control on North Atlantic ε_Nd_ evolution (*58*).

### Implications for glacial NE Atlantic trace element cycling

Our results, together with those of Roberts and Piotrowski (*27*), demonstrate that Iceland exerted a strong and systematic control on the isotopic composition of glacial NE Atlantic waters feeding GNAIW. Although the ODP 982 record represents only the upper GNAIW, the BOFS cores covering the entire 1000- to 4000-m water column, albeit restricted to the past 40,000 years, show the same pattern. Together, these datasets indicate that much of the NE Atlantic water column became more radiogenic during glacial periods, in phase with global ice growth and decay. This Icelandic Ice Sheet–regulated radiogenic input therefore represents a key, previously left unnoticed component of the glacial northern end-member. If ice sheet–modulated Iceland inputs exerted such a strong control on seawater ε_Nd_ in the NE Atlantic, similar processes are likely to have operated in other high-latitude ice-sheet systems. This view is supported by observations from the Norwegian Sea, where seawater ε_Nd_ shifted toward less negative values of −12 to −15 during the LGM (Fig. 4E and Table 1), consistent with enhanced weathering inputs from the Svalbard-Barents Ice Sheets (*25*). A comparable signal is observed in the Labrador Sea, where bottom-water ε_Nd_ values reached −16 ± 1 during the LGM (Fig. 4C) (*56*). While this negative shift has been attributed to the influence of water sourced from Nordic Seas, it may also reflect contributions from Laurentide Ice Sheet. These observations motivate the hypothesis that glacial GNAIW was not isotopically homogeneous but instead comprised at least two regional end-members (*78*): a more radiogenic eastern component influenced by Icelandic volcanic inputs and a less radiogenic western component shaped by Laurentide and Greenland ice sheet contributions. Such east-west heterogeneity provides a coherent framework for reconciling ε_Nd_ distribution in the glacial NE Atlantic and Labrador Sea, as well as GNAIW ε_Nd_ values of ∼−10 in the mid-depth North Atlantic (*24*), reflecting mixing of eastern and western GNAIW components during southward advection. We explore the plausibility of this hypothesis using sensitivity experiments with the Global Neodymium Ocean Model (GNOM) (*79*). In a first experiment, we prescribe a radiogenic NSW mass (ε_Nd_ = −7) in the NE Atlantic. This experiment yields ε_Nd_ values of −9.0 to −8.5 in the mid-depth and deep Atlantic (fig. S7), more radiogenic than the reported GNAIW benchmark value of ∼−10 (*24*), implying the presence of an additional less radiogenic Nd source counterbalancing the Icelandic contribution. To account for this missing component, we conduct a second experiment in which we introduce a strongly unradiogenic NSW mass (ε_Nd_ = −28) in Baffin Bay, representing enhanced erosion and weathering associated with the Laurentide and Greenland ice sheets, supported by the presence of Archean-aged Laurentian detrital carbonates in the North Atlantic region, which constitute a highly unradiogenic and potentially reactive Nd source (*56*). Under these combined eastern-western boundary conditions, GNOM reproduces intermediate ε_Nd_ values of ∼−10.5 to −9.5 in the mid-depth and deep Atlantic (fig. S8), consistent with sedimentary reconstructions (*3*, *21*, *24*). Although these experiments are intentionally simplified and rely on modern circulation geometry and present-day source-sink parameterizations (see the Supplementary Materials for details), they demonstrate that the coexistence of a radiogenic eastern GNAIW component and an unradiogenic western component offers a physically plausible mechanism for explaining downstream ε_Nd_ signatures during glacial periods.

This revised perspective may reshape the interpretation of seawater ε_Nd_ records also from the South and deep Atlantic. Previous studies of deep Atlantic sites (e.g., IODP U1313 at 3414 m, ODP 1063 at 4584 m, and GGC 17 at 5010 m; Figs. 2E and 4D) document a shift to more positive ε_Nd_ values (∼−10) during glacials, which has typically been interpreted as a reduction in NSW production and an enhanced influence of SSW (*3*, *4*, *80*). However, if the glacial NSW itself was relatively more positive because of the Icelandic inputs, these deep-Atlantic data can equally reflect a substantial persistence of NSWs. Independent constraints also indicate that NSW contributed more than 50% to the deep North Atlantic during the LGM (*81*). Therefore, the pervasive radiogenic ε_Nd_ signature of the glacial Atlantic likely reflects a combination of both a changed northern end-member and sustained NSW mass proportions, rather than its wholesale replacement by SSW.

In view of the potentially large Nd contributions from Iceland systems revealed in our study, particularly during glacial periods, we quantified the Nd flux from Iceland via a simple steady-state box model (fig. S9). The model was constrained by a mean glacial GNAIW ε_Nd_ value of −7.2 and a mean Icelandic end-member value (ε_Nd_ = +6), with Atlantic inflow characterized by ε_Nd_ = −14.5, similar to modern conditions (*12*, *82*). Assuming a water-mass transport of 10 sverdrups, representative of typical glacial GNAIW volume transport (*83*), this yields an Icelandic Nd input flux of ∼3.9 × 10^8^ g year^−1^. To further evaluate the uncertainty of this estimate, we propagated plausible end-member ranges of NE Atlantic surface seawater ε_Nd_ (−16.0 to −10.0) in glacial and Icelandic source ε_Nd_ (+4 to +8), together with a bracketing range of effective water-mass transport (5 to 10 sverdrups), using 20,000 Latin hypercube samples. The resulting flux distribution gives a median Icelandic Nd flux of 2.4 [1.0, 4.8] × 10^8^ g year^−1^ [95% confidence interval (CI)] (fig. S10). A key uncertainty in the calculation is the effective transport of NSWs that acquired an Icelandic radiogenic imprint before being exported southward. Because the fractional contribution of the eastern component cannot be independently constrained, we do not assume that the full glacial GNAIW transport experienced Icelandic modification. Instead, we bracket the effective transport through the Iceland-influenced formation/exchange region by 5 to 10 sverdrups. The upper bound (10 sverdrups) corresponds to a typical estimate for glacial GNAIW transport, whereas the lower bound (5 sverdrups) reflects the likelihood that only a subset of this transport was ventilated and substantially modified in the NE Atlantic close to Iceland. Notably, radiogenic ε_Nd_ values (−8 to −4) observed across much of the NE Atlantic water column (1000 to 4000 m) during glacial periods imply that our intermediate water–based flux estimates are conservative lower bounds. The derived flux of 2.4 [1.0, 4.8] × 10^8^ g year^−1^ is comparable to the estimated global dust Nd input (2 × 10^8^ to 4 × 10^8^ g year^−1^) and represents 2.7% [1.1 to 5.3%] of the global Nd input to the oceans of 9 × 10^9^ g year^−1^ or 4.4% [1.8 to 8.7%] if a smaller global input of 5.5 × 10^9^ g year^−1^ is adopted (*6*, *84*, *85*). Although the glacial global Nd budget remains uncertain, this magnitude indicates that the Icelandic flux constituted an important component of the glacial North Atlantic Nd cycle.

The strong coupling among Icelandic Ice Sheet dynamics, volcanic Nd input, and seawater ε_Nd_ evolution has further implications for trace element cycling in the glacial NE Atlantic. Enhanced subglacial erosion and comminution of basaltic bedrock during glacial maxima likely produced highly reactive detritus rich in iron (Fe), manganese (Mn), and other micronutrients, which would have been released to surrounding waters during partial dissolution (*71*). This interpretation agrees with modern evidence showing that ice-sheet systems are important sources of both macronutrients [nitrogen (N), phosphorus (P), and silicon (Si)] and micronutrients such as Fe (*86*–*89*). Although the net effect of such inputs on primary productivity depends on additional controls including macronutrient availability, light, and temperature, reconstructed LGM productivity south of Iceland and off southern Greenland is comparable to modern values in both summer and winter (*90*). Constraining the coupled processes of ice-sheet dynamics and oceanic trace element cycling is therefore essential for reconstructing glacial trace element and isotope budgets and for assessing their role in modulating nutrient availability and climate feedbacks in the North Atlantic.

## MATERIALS AND METHODS

### Core information and chronology

ODP Site 982 was drilled in 1995 during Leg 162 of the Ocean Drilling Program. The site lies on the Rockall Plateau in the eastern North Atlantic (57°30.992N, 15°52.001W; 1135-m water depth) between the Halton Bank and Rockall Bank (Shipboard Scientific Party, 1996). Sediment samples from Hole A were taken in 5- to 10-cm intervals. Chronological control was established by tuning benthic foraminiferal δ^18^O (*Cibicidoides* spp.) to the global LR04 (*91*, *92*). To assess how uncertainties in this tuning propagate into the Nd isotope record, we developed an MC framework (3000 realizations) that incorporates (i) analytical δ^18^O errors (±0.18‰), (ii) sampling-resolution limitations evaluated against high-frequency LR04 variability, and (iii) ±1-cm depth uncertainties in Nd sampling resulting from repeated historical sampling and foam refilling of the core. This ensemble approach highlights that the δ^18^O measurement error dominates during steep glacial-interglacial transitions, whereas coarse sampling resolution contributes the most in flat δ^18^O intervals (e.g., interglacials). As a result, tie-point age uncertainties are typically ∼1 to 2 ka, and Nd samples carry broader uncertainties of ∼2 to 4 ka (95% CI). This probabilistic age-depth framework ensures that our interpretations of Nd isotope variability are grounded in a robust assessment of chronological uncertainty. Full methodological details are provided in the Supplementary Materials (fig. S2).

### Sample treatment

Sediment samples were freeze dried and homogenized before weighing. Approximately 0.4 to 0.5 g of sediment was treated first with >18.2-MΩ grade deionized water to remove any exchangeable ions (*93*). Sediment was then treated with a weak reductive solution (0.005 M hydroxylamine hydrochloride/1.5% acetic acid/0.03 M Na-EDTA solution buffered to pH 4 with trace metal clean class ammonia) for 10 s following Huang *et al.* (*93*) for extracting the authigenic Fe-Mn oxyhydroxide fraction. Total procedural blanks were below 0.25 ng for Nd (*n* = 6), which is well below 0.1% of sample yields, and were hence negligible. The reproducibility was monitored by leaching two repeat samples with ε_Nd_ values of −12.0 ± 0.16 (±2 SD, *n* = 3) and −10.9 ± 0.05 (±2 SD, *n* = 4), respectively. Carbonate was removed using a Na acetate solution buffer to pH 4 with acetic acid (Merck Suprapur) following Gutjahr *et al.* (*94*). These sequential extraction steps do not dissolve silicate phases; any reactive Icelandic basaltic material present in the sediment would therefore be retained in the residual fraction. After centrifugation and decanting of the supernatant, the sediment sample was triple rinsed in >18.2-MΩ water. The sediments were then treated with a strong reductive solution (0.05 M hydroxylamine hydrochloride/15% acetic acid/0.03 M Na-EDTA solution buffered to pH 4 with trace metal clean class ammonia) overnight to completely remove residual Fe-Mn oxyhydroxides following the method applied by Gutjahr *et al.* (*94*). The residual samples were dried at a low temperature (<45°C) in an oven and homogenized before alkaline fusion following Bayon *et al.* (*95*). To obtain the Nd isotopic signature of clay-size residual samples, several same depth/age samples were selected and treated with the same procedure above (i.e., deionized water, weak reductive solution, and decarbonated, strong reductive solution) but separated into clay and silt fractions following the method of Bretschneider *et al.* (*96*) before alkaline fusion. The accuracy and reproducibility of the fusion technique were monitored by processing reference materials including the USGS reference material BHVO-2 (*n* = 3) and repeat sediment samples, as shown in data S1. Total procedural blanks were below 0.3 ng for Nd (*n* = 3) and are therefore negligible.

### Nd isotope analyses

The REEs of samples were separated from matrix elements using cation exchange chromatography (AG 50W-X8) following the scheme of Stichel *et al.* (*14*). Nd was further separated from the other REEs for isotope measurements using Eichrom LN-Spec resin following the procedure of Pin and Zalduegui (*97*). The ^143^Nd/^144^Nd ratios were measured on a Neptune Plus MC-ICP-MS at the Institute of Environmental Physics of Heidelberg University and were corrected for instrumental mass bias to ^146^Nd/^144^Nd = 0.7219 following the approach of Vance and Thirlwall (*98*). During the measurement, the normalized argon index was monitored and was higher than 0.25 to obtain a hot plasma for decreasing the nonexponential mass fractionation of Nd analyses following Yu *et al.* (*99*). The ^143^Nd/^144^Nd ratios of all samples were normalized to bracketing analyses of the GSB 04-3258-2015 standard with a value of 0.512438 (*100*). The secondary standard solution NIST 3135a, USGS reference material NOD-A-1, and internal reference HeiNds were run with authigenic samples, and the USGS reference material BHVO-2 was run with residual samples to check the accuracy and external reproducibility of the procedure for Nd isotope measurements (data S1). The external reproducibility of the Nd isotope measurements of samples was determined using standard solutions with concentrations matching those of the measured samples in the range of 0.15 to 0.18 ε_Nd_ units (2 SD). The Nd isotopic composition is expressed as ε_Nd_ = [(^143^Nd/^144^Nd)_sample_/(^143^Nd/^144^Nd)_CHUR_ − 1] × 10^4^ with CHUR (chondritic uniform reservoir) = 0.512638 (*101*). The pooled 2 SD of the secondary standard NIST, NOD-A-1, and HeiNds is used to illustrate the reproducibility of measured Nd isotopic compositions in all figures and is shown in data S1.

### Forward mixing model for NE Atlantic ε_Nd_ variability

We developed a forward mixing model with MC uncertainty propagation to reconstruct NE Atlantic ε_Nd_ variability and quantify the relative contribution of Iceland-sourced Nd. The model is physically constrained by the LR04 benthic δ^18^O stack and calibrated to the LGM ε_Nd_ value of −7.7 at ODP Site 982, incorporating both deterministic relationships and comprehensive uncertainty propagation. In the first stage, we established a deterministic LR04-based projection that translates the LR04 stack into Icelandic flux variations. We defined a glacial index *S*(*t*) through min-max scaling of the δ^18^O record (Eq. 1)

$$S\left(t\right)=\frac{\delta \left(t\right)-\delta \left(\text{min}\right)}{\delta \left(\text{max}\right)-\delta \left(\text{min}\right)}\in \left[0,1\right]$$ (1)

The raw Icelandic fraction *F*_raw_(*t*) was set equal to *S*(*t*), with a scaling factor λ determined to reproduce the target LGM ε_Nd_. End-member values were based on modern water mass characteristics: ε_bg_ = −13.8 (NE Atlantic Seawater at ∼1000-m depth) and ε_I_ = +6 (mean value of Icelandic input). The predicted ε_Nd_ [ε_pred_(*t*)] follows below mixing (Eq. 2)

$$\varepsilon_{\text{pred}}\;\left(t\right)=\varepsilon_{\text{bg}}\left[1-\lambda F_{\text{raw}}\left(t\right)\right]+\varepsilon_{I}\lambda F_{\text{raw}}\left(t\right)$$ (2)

To capture suborbital variability not explained by simple ice-volume forcing, we implemented a time-dependent modulation scheme *M*(*t*) applied to *F*_raw_ before λ-scaling. This dual modulation incorporates time-variable transport efficiencies: (i) buffering during 30 to 120 ka primarily weighted by the δ^18^O-derived ice-growth rate (85%) with a secondary absolute-δ^18^O term (15%) and (ii) enhancement during 170 to 195 ka with similar weighting. Fast-glaciation events (growth rates in top 30%, δ^18^O ≥ 4.0‰, duration ≥1 ka) received special treatment: protection from excessive attenuation during buffering and amplification during enhancement. The effective Icelandic fraction [*F*(*t*)] becomes (Eq. 3)

$$F\left(t\right)=\text{clip}\;\left\{\lambda \cdot \text{clip}\left[F_{\text{raw}}\left(t\right)\;M\left(t\right),\;0,\;1\right],0,1\right\}$$ (3)

In the second stage, we propagated uncertainties through an MC ensemble (*N* = 2000). Each realization incorporated Nd age uncertainties, Icelandic end-member variability (ε_I_ = +4 to +8), and state-dependent background ε_Nd_ (interglacials: −13.8; glacials: −16.0 to −10.0). Ensemble predictions were interpolated to original Nd ages and summarized as P10/P50/P90 percentiles. Model performance was evaluated using both *R*^2^ and RMSE between the median predictions and observations. Further parameter ranges and details are provided in the Supplementary Materials (tables S1 to S3 and fig. S4).
