# Supplementary material for: Ice-sheet dynamics drive glacial-interglacial shifts in North Atlantic seawater neodymium isotopes
Source: Sci Adv. 2026 Aug 19;12(34):eaeg5747. doi: 10.1126/sciadv.aeg5747 (PMC13488906; doi:10.1126/sciadv.aeg5747)
Supplement: Supplementary file 1 [file sciadv.aeg5747_sm.pdf]

Science Advances
AAAS

Supplementary Materials for

Ice-sheet dynamics drive glacial-interglacial shifts in North Atlantic seawater neodymium isotopes

Antao Xu *et al.*

Corresponding author: Antao Xu, axu16@outlook.com

Sci. Adv. **12**, eaeg5747 (2026)
DOI: 10.1126/sciadv.aeg5747

The PDF file includes:

Supplementary Text
Figs. S1 to S10
Tables S1 to S4
Legend for data S1

Other Supplementary Material for this manuscript includes the following:

Data S1

Supplementary Text

1. Rare earth elements and major elements concentration analyses

The concentration of REEs and major elements of authigenic Fe–Mn oxyhydroxide were measured on an ICAP Q ICPMS (Thermo Fisher Scientific) at the Institute of Environmental Physics of Heidelberg University. For sample uptake and desolvation, the combination of an SC2-DX Autosampler (ESI) and an APEX HF (ESI) desolvating system was used. The measurement and evaluation protocol closely follow Blaser et al. (*35*). Oxide formation during measurement was monitored with element solutions of barium (Ba), cerium (Ce), praseodymium (Pr) + Nd, and samarium (Sm) + europium (Eu) + gadolinium (Gd) + terbium (Tb) at the start and end of each analytical session. The measurement reproducibility and accuracy were assured by measuring diluted USGS reference material NOD-A-1 solution, replicates and the diluted seawater standard SPSSW2 throughout the measurement sequence. The external reproducibility for each element was typically better than 3% (RSD). Mean values and 2SD for the reference material measurements are given in Supplementary Data 1.

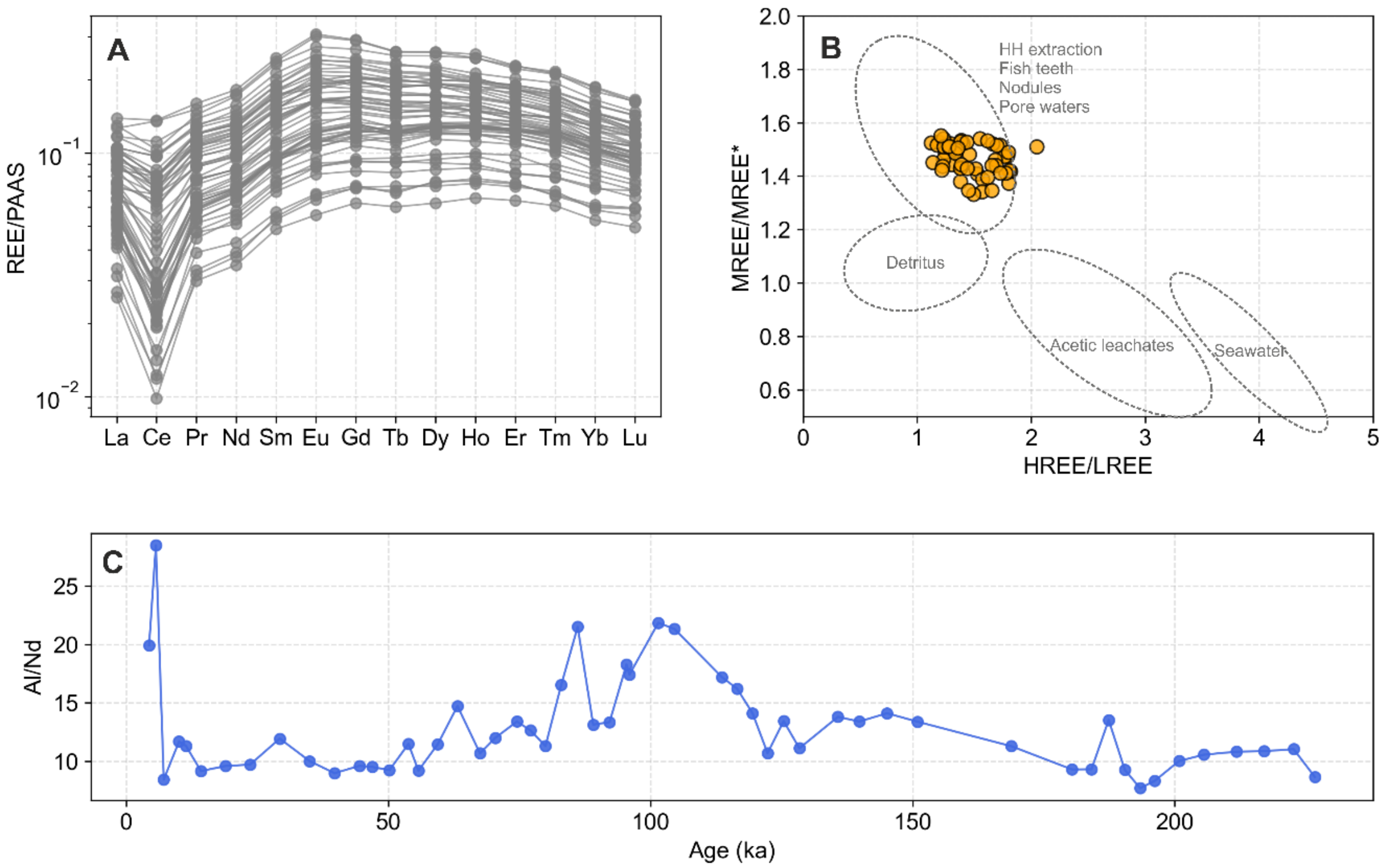

Supplementary Fig. S1. REE patterns and elementary ratios of leachates. (**A**) REE patterns for all samples. (**B**) Plot of the MREE bulge against the REE slope for all samples. The dashed-line circles indicate typical ranges for different materials (*36*). (**C**) Aluminum-to-neodymium (Al/Nd) ratios for all samples.

2. Age-depth model uncertainty quantification for ODP Site 982

We developed an improved Monte Carlo framework to quantify age-depth uncertainties for ODP Site 982 by combining tie-point errors, sampling-resolution effects, and $\delta^{18}O$ measurement uncertainties. The method extends standard tie-point resampling approaches by explicitly incorporating high-frequency variability in the LR04 benthic $\delta^{18}O$ stack to better constrain the sampling-resolution component of age uncertainty. Benthic $\delta^{18}O$ data for the LR04 global stack are from Lisiecki and Raymo (*91*) and ODP 982 benthic $\delta^{18}O$ (*Cibicidoides* spp.) data are from Venz et al. (*92*). The model treats the LR04 $\delta^{18}O$ stack as the reference chronostratigraphic framework.

2.1 Calculation of local LR04 slopes

To convert $\delta^{18}O$ measurement errors into time-domain uncertainties, the local slope of the LR04 curve ($\delta^{18}O$ vs. age) was estimated using a Savitzky–Golay filter (window length=21 points; polynomial order=2). The smoothed curve was numerically differentiated with respect to age (ka) to obtain a local slope at each tie-point, thereby capturing the rate of isotopic change through time.

2.2 Improved sampling-resolution error estimation

Traditional tie-point uncertainty estimates assume a constant interpolation error proportional to the tie-point spacing ($\Delta t/\sqrt{12}$). However, this underestimates uncertainty during intervals of rapid isotopic variability. We therefore introduced a new "variability-based" sampling-resolution term derived from the local high-frequency structure of the LR04 record. For each ODP tie-point age (t_i), we computed the 95% $\delta^{18}O$ range (2.5-97.5th percentile) within a ±10 ka window around (t_i), denoted as $\Delta\delta^{18}O_{95}$. This variability range was converted into a time uncertainty using the local slope (Eq. 1, Eq. 2).

$$si = \frac{d(\delta^{18}O)}{dt} \text{ (Eq. 1)}$$

$$\sigma_{var,i} = 0.5 \times \frac{\Delta(\delta^{18}O_{95})}{|si|} \text{ (Eq. 2)}$$

A conservative cap of 2 ka was applied to avoid unrealistically large uncertainties in intervals of near-zero slope. The final sampling-resolution uncertainty for each tie-point was defined as:

$$\sigma_{sampling,i} = \max(\sigma_{interp,i}, \sigma_{var,i}) \text{ (Eq. 3)}$$

where $\sigma_{interp,i} = \Delta t_i/\sqrt{12}$ is the traditional interpolation error.

2.3 Conversion of $\delta^{18}O$ measurement uncertainty to time-domain error

For each tie-point, the reported analytical $\delta^{18}O$ uncertainty ($\sigma\delta$, ±0.18‰) was propagated into an age uncertainty (σ_t) using the LR04 local slope:

$$\sigma_{\delta^{18}O,i}^{time} = \frac{|\sigma_{\delta,i}|}{|si|} \text{ (Eq. 4)}$$

To prevent division by small slopes, σ_t values were capped at 2 ka.

2.4 Total tie-point uncertainty

The total age uncertainty at each tie-point was calculated as the quadratic sum of the sampling-resolution and $\delta^{18}O$-derived components:

$$\sigma_{t,i} = \sqrt{(\sigma_{sampling,i})^2 + (\sigma_{\delta^{18}O,i}^{time})^2} \text{ (Eq. 5)}$$

2.5 Monte Carlo propagation

A Monte Carlo ensemble (n=3000 realizations) was generated by random sampling of tie-point ages from Gaussian distributions. For each realization, an independent age-depth model was created by linear interpolation between perturbed tie-points. To account for sample depth uncertainty, each ε_{Nd} sample depth was perturbed by either a uniform or normal random offset (±1cm depth error) and converted to age via interpolation within each Monte Carlo age–depth model. This process yielded an ensemble of 3000 age estimates per ε_{Nd} sample.

2.6 Uncertainty decomposition and validation

To quantify the contribution of each error source, the total age uncertainty was decomposed into variance fractions attributable to sampling resolution and $\delta^{18}O$ measurement:

$$f_{sampling} = \frac{\sigma_{sampling}^2}{\sigma_t^2}, f_{\delta^{18}O} = \frac{(\sigma_{\delta^{18}O,i}^{time})^2}{\sigma_t^2} \text{ (Eq. 6)}$$

Additional validation steps were implemented: 1) the LR04 curve was resampled at ODP tie-point spacing 1000 times to quantify root-mean-square errors relative to the original high-resolution stack, yielding a 95th-percentile RMS of typically <0.1 ‰; 2) the LR04 power spectrum was compared to the Nyquist frequency of ODP 982 sampling to assess the fraction of unresolved high-frequency variance, which was empirically related to potential age errors (<0.5 ka for most intervals).

The improved Monte Carlo framework yields average 1σ tie-point age uncertainties of ~1-1.5 ka and ε_{Nd} sample age uncertainties of ~2 ka. Variance decomposition indicates that sampling-resolution variability accounts for ~60-70% of the total age variance, while $\delta^{18}O$ measurement

contributes ~30-40%. The resulting ensemble provides a robust quantitative basis for propagating chronological uncertainty into the ε_{Nd} time series (fig. S2).

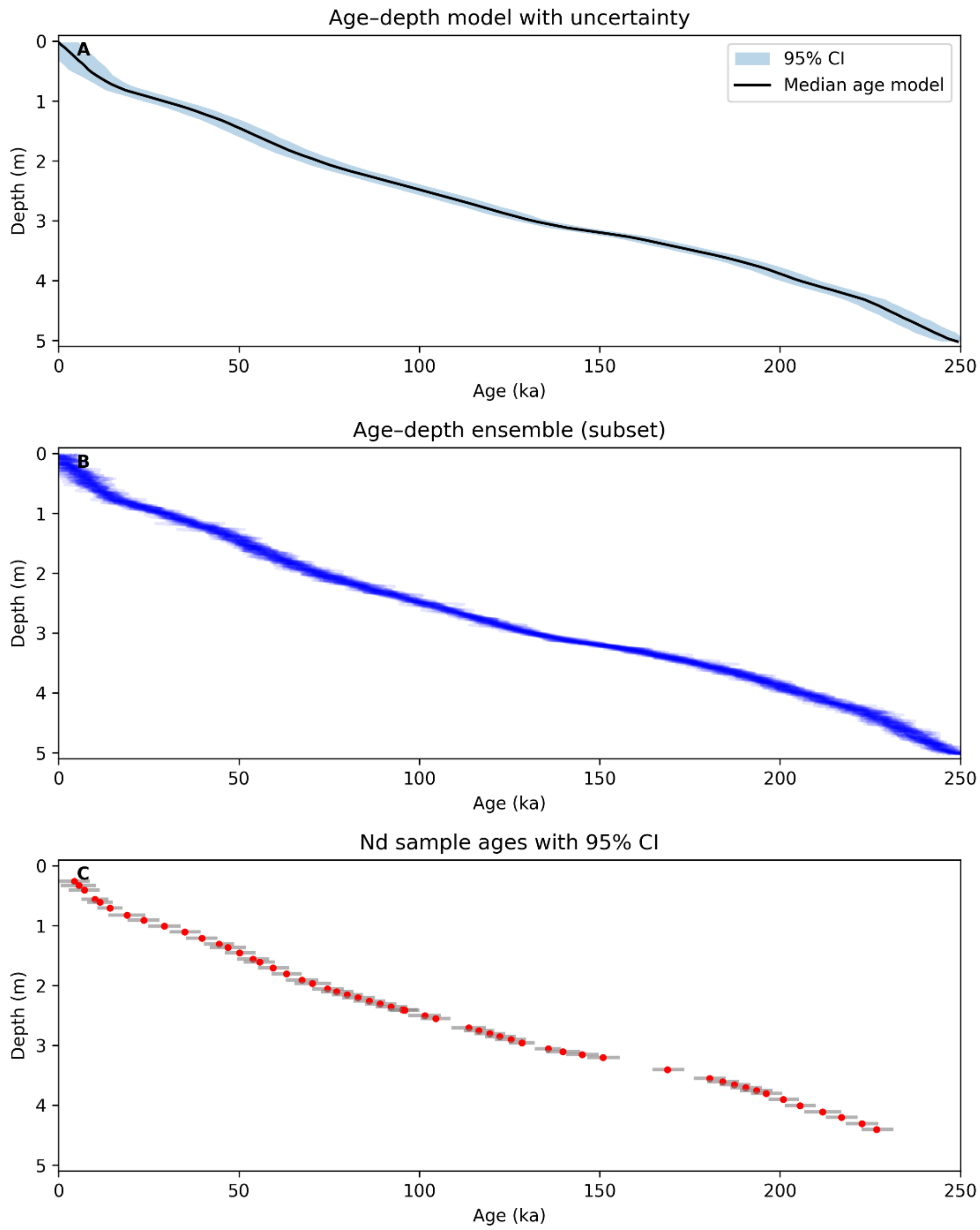

Supplementary Fig. S2. Enhanced age-depth analysis for ODP 982 with propagated age uncertainties. (**A**) Median age-depth model (black line) and its 95% confidence envelope (shaded) derived from Monte Carlo propagation of tie-point uncertainties, where $\delta^{18}O$ measurement errors are converted to time using the local LR04 slope and combined with an LR04 variability-based sampling-resolution term. (**B**) A random subset of ensemble realizations (up to 50) illustrating the spread of plausible age-depth trajectories consistent with the constraints. (**C**) Nd sample ages with horizontal bars showing 95% CIs and red symbols marking the median ages, referenced to the same age-depth solution. The panels demonstrate that the improved uncertainty scheme yields a well-constrained, internally consistent age framework for ODP 982 and quantitatively brackets the chronological uncertainty carried into the ε_{Nd} record.

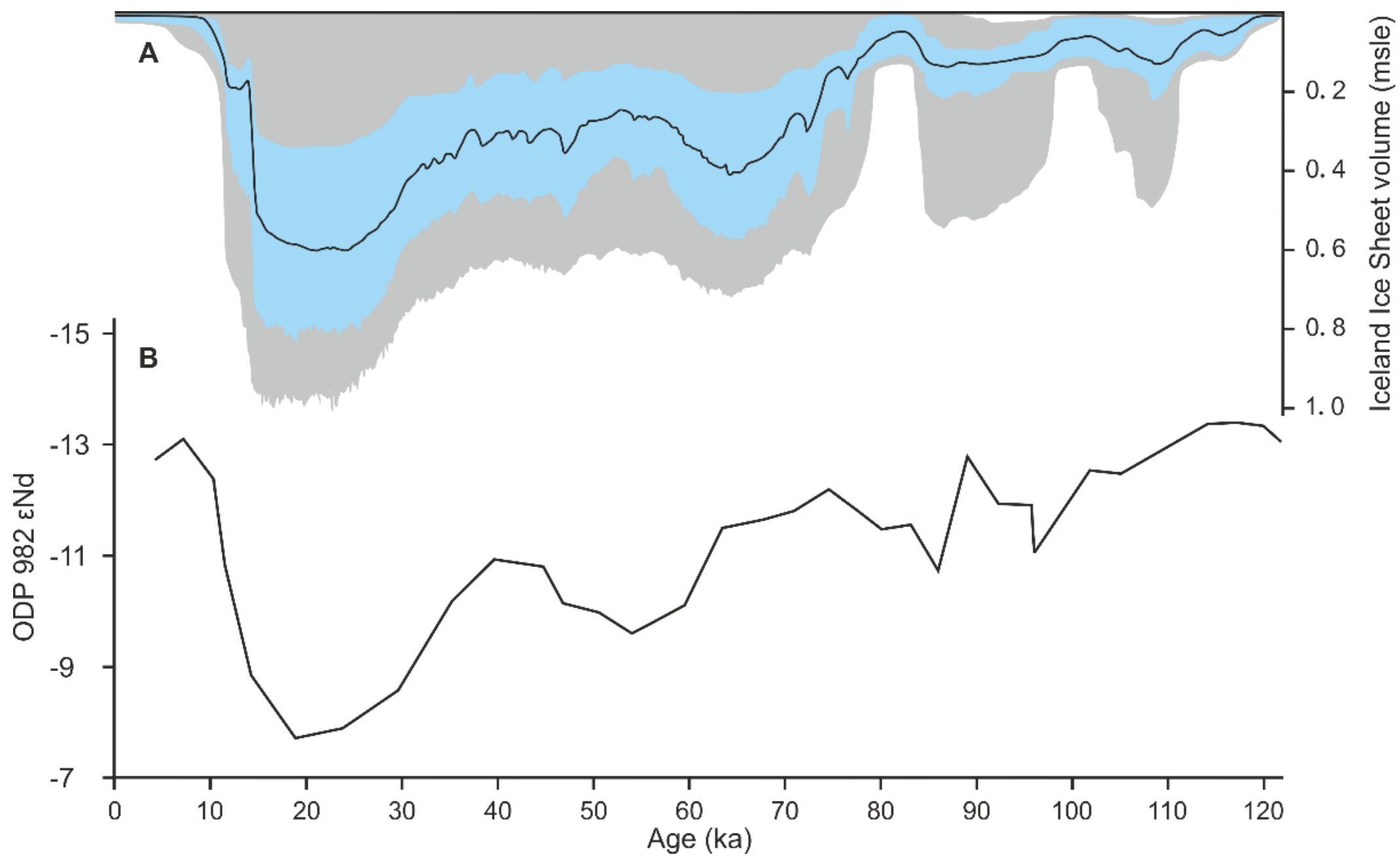

Supplementary Fig. S3. Time series of the reconstructed Icelandic Ice Sheet (IIS) volume over the last glacial cycle based on Glacial Systems Modeling (*70*) (A) and the ODP 982 ε_{Nd} record (B). In panel a, the gray shaded area represents the full ensemble, whereas the blue shading denotes the sieved "non-ruled-out-yet" (NROYtier1) sub-ensemble. The black curve shows the median NROYtier1 solution.

3. Forward mixing model for NE Atlantic ε_{Nd} variability

We reconstruct NE Atlantic ε_{Nd} variability and the relative Icelandic contribution using a physically anchored forward model comprising three components: (1) an LR04-based projection that maps $\delta^{18}O$ to an Icelandic fraction; (2) a time-dependent modulation reflecting buffered and enhanced intervals with safeguards for fast glaciation; and (3) a Monte Carlo (MC) ensemble with track-specific LGM anchoring to propagate chronological and endmember uncertainties.

3.1 Notation

Supplementary Table. S1. Symbols used in the Methods

Symbol	Meaning	Symbol	Meaning
$\delta(t)$	LR04 benthic $\delta^{18}O$ at age t	$F_{raw}(t)$	Raw Icelandic fraction before modulation
$S(t)$	Glacial index (min–max normalized $\delta^{18}O$)	$M(t)$	Time-dependent modulation factor
$r(t)$	Ice-growth rate	$F(t)$	Final Icelandic fraction after modulation & LGM scaling
$\varepsilon_{obs}(t_i)$	Observed ε_{Nd} at ODP 982, age t_i	λ	LGM scale factor
$\varepsilon_{pred}(t)$	Predicted ε_{Nd}	C	Cold set $\{t: S(t) \geq S_{0.95}\}$
ε_{bg}	NE Atlantic seawater ε_{Nd}	P10/P50/P90	10th/50th/90th ensemble percentiles
ε_I	Icelandic ε_{Nd} endmember		

3.2 LR04-anchored forward model

3.2.1 Glacial index and raw Icelandic fraction

We map $\delta^{18}O$ to a unitless glacial index by min–max scaling:

$$S(t) = \frac{\delta(t) - \delta(min)}{\delta(max) - \delta(min)} \in [0, 1] \quad \text{(Eq. 7)}$$

This scaling maps the full $\delta^{18}O$ range (approximately 3.0-5.0‰) to the interval [0,1], with higher values indicating more glacial conditions.

Define the cold set by the 95th percentile of S:

$$C = \{t : S(t) \geq S_{0.95}\} \quad \text{(Eq. 8)}$$

The raw Icelandic flux fraction was set proportional to the glacial index:

$$F_{raw}(t) = S(t) \quad \text{(Eq. 9)}$$

3.2.2 Time-dependent modulation

(1) Buffering window

Before the LGM scale is applied, we modulate the Icelandic fraction:

$$F_{mod}(t) = clip(F_{raw}(t) M(t), 0, 1) \quad \text{(Eq. 10)}$$

Within $30 \leq t \leq 120$ ka, combine two piecewise-linear maps: 1) rate-based damping $m_r \in [0.25, 1.00]$ by mapping $r(t)$ from [0.01, 0.05] ‰ kyr^{-1} to [0.25, 1.00] (clamped); 2) $\delta^{18}O$-based damping $m_\delta \in [0.60, 1.00]$ by mapping $\delta(t)$ from [3.5, 4.0] ‰ to [0.60, 1.00]. Weighted sum:

$$M(t) = 0.85 M_r(t) + 0.15 M_\delta(t) \quad \text{(Eq. 11)}$$

If flagged as fast glaciation (see below), enforce $M(t) \geq 0.8$.

(2) Enhancement window

Rate-based enhancement $m_r \in [1.00, 2.50]$ across [0.01, 0.05] ‰ kyr^{-1} $\delta^{18}O$ co-term $m_\delta \in [1.00, 1.50]$ across [3.5, 4.0] ‰. Weights 0.8/0.2. If also fast glaciation, apply an extra ×1.5. A centered 4-point moving average smooths $M(t)$.

3.3 LGM anchoring and mixing

Deterministic endmembers use $\varepsilon_{bg} = -13.8$ and $\varepsilon_I = +6$. The LGM target is $\varepsilon_{LGM} = -7.7$. The target fraction is

$$F_{tgt} = clip(\frac{\varepsilon_{LGM} - \varepsilon_{bg}}{\varepsilon_I - \varepsilon_{bg}}, 0, 1) \quad \text{(Eq. 12)}$$

$$\lambda = min(\frac{F_{tgt}}{F_{raw C}}, 1) \quad \text{(Eq.13)}$$

Final fraction:

$$F(t) = clip\,(\lambda . clip(F_{raw}\,(t)\,M(t),\ 0,\ 1), 0, 1) \quad \text{(Eq. 14)}$$

Predicted ε_{Nd}:

$$\varepsilon_{pred}(\mathrm{t}) = \varepsilon_{bg}\,[1 - F(t)] + \varepsilon_I F(t) \quad \text{(Eq. 15)}$$

3.4 Fast-glaciation detection

We smooth $\delta^{18}O$ and compute:

$$\mathrm{r(t)} = -\frac{\mathrm{d}_\delta}{\mathrm{d}_t} \quad \text{(Eq. 16)}$$

A time is flagged fast glaciation if: (i) $r(t)$ lies in the top 30% of positive rates, (ii) $\delta(t)$ ≥ 4.0 ‰, and (iii) the contiguous duration is ≥1 ka.

3.5 Monte Carlo ensemble (uncertainty propagation)

We conducted 2000 Monte Carlo realizations to quantify uncertainties from age and endmember variability (table S2 and S3). For each realization k:

(1) Age jitter. $t_i^{(k)} \sim N(t_i, \sigma_{ti}^2)$.

(2) Endmembers. $\varepsilon_I^{(k)} \sim U\,(+4, +8)$. Background vector:

$$\varepsilon_{bg}^{(k)}(\mathrm{t}) = \begin{cases} -13.8, & \delta(t) < 4.0‰ \\ u(-14.5, -10.0), & \delta(t) \geq 4.0‰ \end{cases} \quad \text{(Eq. 17)}$$

(3) Modulation and fraction. Evaluate $M^{(k)}(t_i^{(k)})$; compute $F^{(k)}(t)$ by Eq. 14.

(4) Track-specific LGM anchoring. Using the coldest 5% (by S) and the mean background across those times:

$$\mathrm{F}_{tgt}^{(k)}(t) = clip\left(\frac{\varepsilon_{LGM} - \overline{\varepsilon_{bg}^{(k)}}}{\varepsilon_I^{(k)} - \overline{\varepsilon_{bg}^{(k)}}}, 0,\ 1\right), \lambda^{(k)} = min\left(\frac{F_{tgt}^{(k)}}{F_{mod}^{(k)}}, 1\right) \quad \text{(Eq. 18)}$$

(5) Mixing and interpolation.

$$\varepsilon_{pred}^{(k)}(t_i^{(k)}) = \varepsilon_{bg}^{(k)}\left(t_i^{(k)}\right)\left[1 - F^{(k)}\right] + \varepsilon_I^{(k)} F^{(k)} \quad \text{(Eq. 19)}$$

(6) Ensemble summaries.

Results report ε_{Nd} and F at P10/P50/P90 across the original Nd age model. Model skill is evaluated using RMSE and R^2 between P50 predictions and observations. Observed versus predicted ε_{Nd} based on the LR04-derived baseline is shown in fig. S4, and observed versus MC median ε_{Nd} with asymmetric uncertainty (P10–P90) and 2SD analytical errors is shown in Fig. 6.

3.6 Parameters and priors

Supplementary Table. S2. Deterministic configuration

Parameter	Symbol and value	Description
Icelandic endmember	$\varepsilon_I = +6$	radiogenic Nd source
Background ε_{Nd}	ε_{bg}= -13.8	NE Atlantic seawater
LGM target ε_{Nd}	ε_{LGM}= -7.7	Observed
Buffer window	30–120 ka	
Enhancement window	170–195 ka	
Rate thresholds	$r_{low} = 0.01$, $r_{high} = 0.05$ ‰ kyr^{-1}	piecewise maps
Buffer damping (rate)	$m_r \in [0.25, 1.00]$	
Buffer damping ($\delta^{18}O$)	$m_\delta \in [0.60, 1.00]$	
Buffer weights	$w_r = 0.85$, $w_\delta = 0.15$	
Safeguard (buffer)	$M \geq 0.8$	
Enhancement (rate)	$m_r \in [1.00, 2.50]$	
Enhancement ($\delta^{18}O$)	$m_\delta \in [1.00, 1.50]$	
Enhancement weights	$w_r = 0.8$, $w_\delta = 0.2$	
Fast-glaciation rules	top 30% $r>0$, $\delta^{18}O \geq 4.0$‰, ≥1 ka	
Modulation smoothing	4 points	moving average
λ cap	$\lambda \leq 1.0$	no amplification beyond raw index

Supplementary Table. S3. Monte Carlo configuration

Parameter	Symbol and value	Description
Icelandic endmember	U (+4, +8)	radiogenic Nd source
Background ε_{Nd} (warm)	ε_{bg} = -13.8	if $\delta^{18}O < 4.0‰$
Background ε_{Nd} (cold)	U (-16.0, -10.0)	if $\delta^{18}O \geq 4.0‰$
Realizations	N = 2000	
Random seed	42	
Age jitter	N (t_i, σ_{ti}^2)	

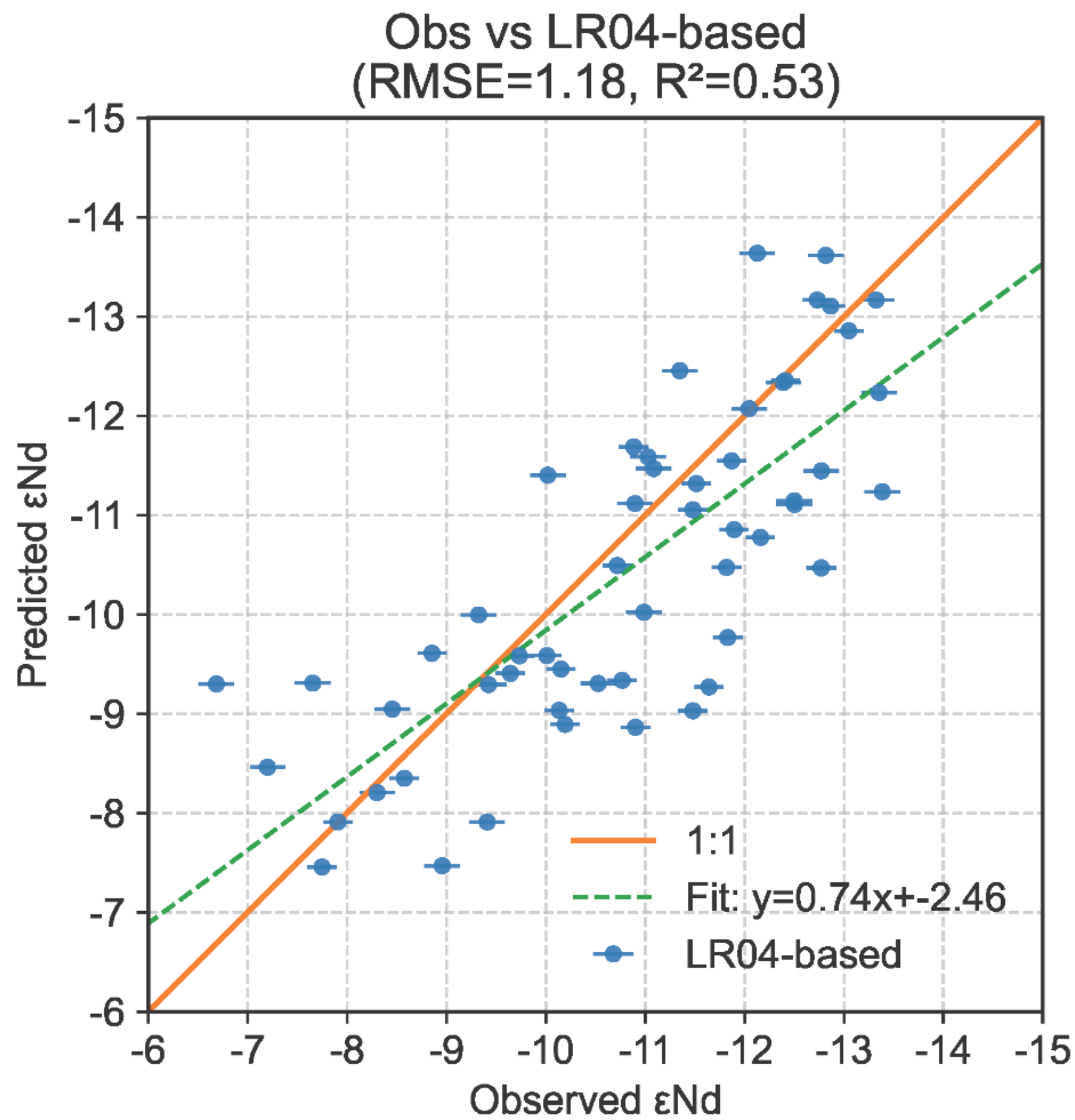

Supplementary Fig. S4 Observed ε_{Nd} versus predicted ε_{Nd} based on LR04-based baseline; the 1:1 line and an ordinary-least-squares fit are shown

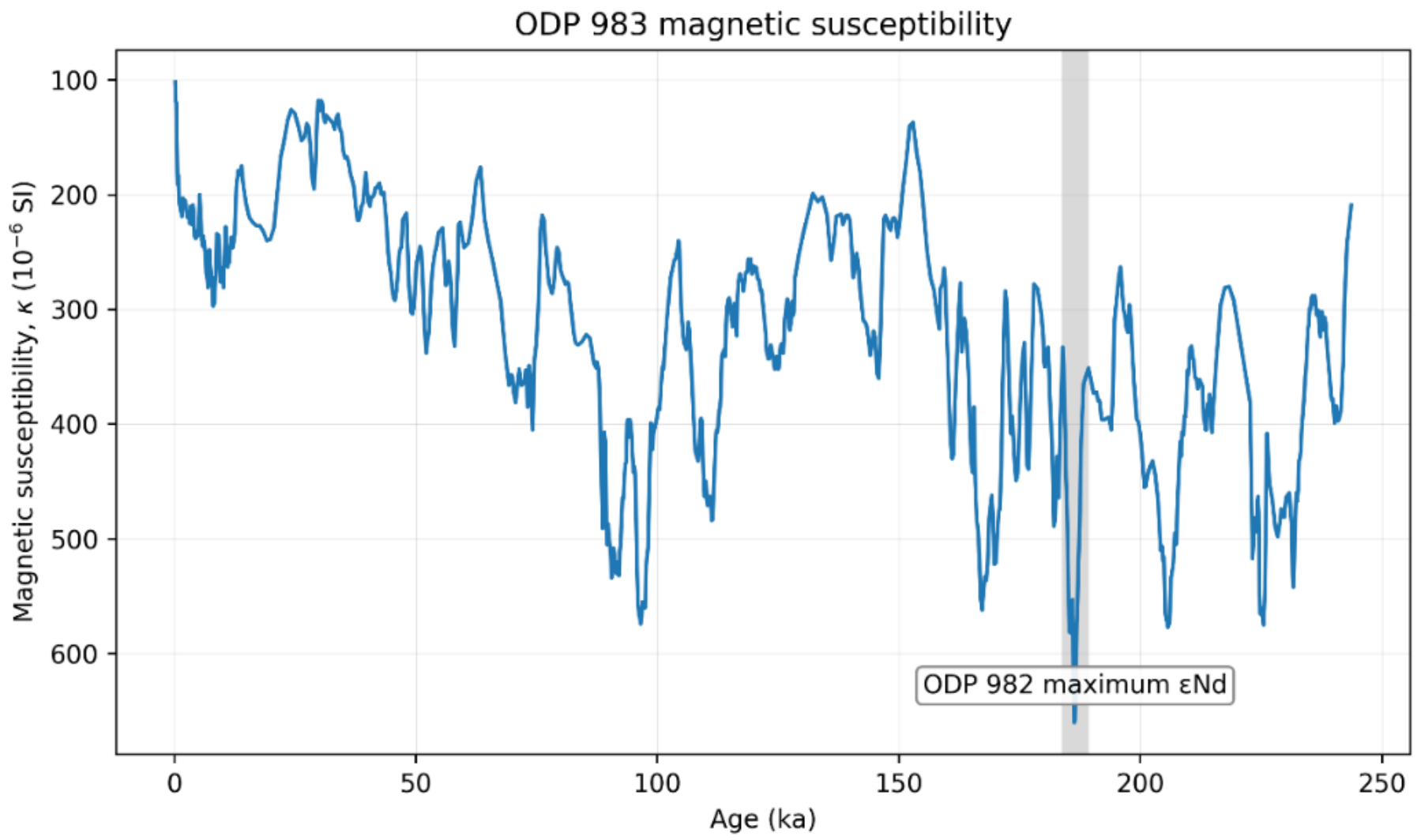

Supplementary Fig. S5 ODP Site 983 magnetic susceptibility record over the past 250 ka, with the interval of maximum ε_{Nd} at ODP Site 982 indicated.

4. Sensitivity experiments with the Global Neodymium Ocean Model (GNOM)

The GNOM is fully described in Pasquier et al. (*79*). Briefly, the model simulates steady-state oceanic Nd cycling under modern circulation, incorporating advection, mixing, reversible scavenging, and benthic sources and sinks. For the sensitivity experiments, two key source regions were defined using comparable grid cells within the model domain: the NE Atlantic and Baffin Bay (fig. S6). In the first experiment, seawater in the NE Atlantic was assigned a fixed ε_{Nd} of -7, representing the radiogenic eastern GNAIW component influenced by Icelandic volcanic inputs as documented at ODP Site 982. All other model parameters, including circulation, Nd concentrations, scavenging, and remaining source/sink terms, were retained at their default modern values. This experiment yields mid-depth and deep Atlantic ε_{Nd} values of -9.0 to -8.5 (fig. S7), more radiogenic than the reconstructed glacial GNAIW benchmark of approximately -10 in the mid-depth North Atlantic (*24*). This positive bias indicates that Icelandic inputs alone produce downstream values that are too radiogenic, suggesting that an additional, less radiogenic Nd source is required to balance the Icelandic contribution. In the second experiment, seawater in Baffin Bay was additionally assigned a fixed ε_{Nd} of -28, representing the unradiogenic western GNAIW component strongly influenced by enhanced erosion and weathering associated with the Laurentide and Greenland ice sheets. Under these combined eastern–western boundary conditions, GNOM reproduces intermediate ε_{Nd} values of approximately -10.5 to -9.5 across the mid-depth and deep Atlantic (fig. S8), consistent with sedimentary reconstructions (*24*). These experiments are intentionally simplified, using modern circulation geometry and present-day source–sink parameterizations, and the prescribed ε_{Nd} values represent idealized endmembers rather than spatially resolved glacial boundary conditions. The results should therefore be interpreted as a first-order diagnostic rather than a glacial simulation. Nevertheless, the sequential comparison demonstrates that the coexistence of a radiogenic eastern and an unradiogenic western GNAIW component offers a plausible mechanism for explaining the observed downstream ε_{Nd} distribution during glacial periods.

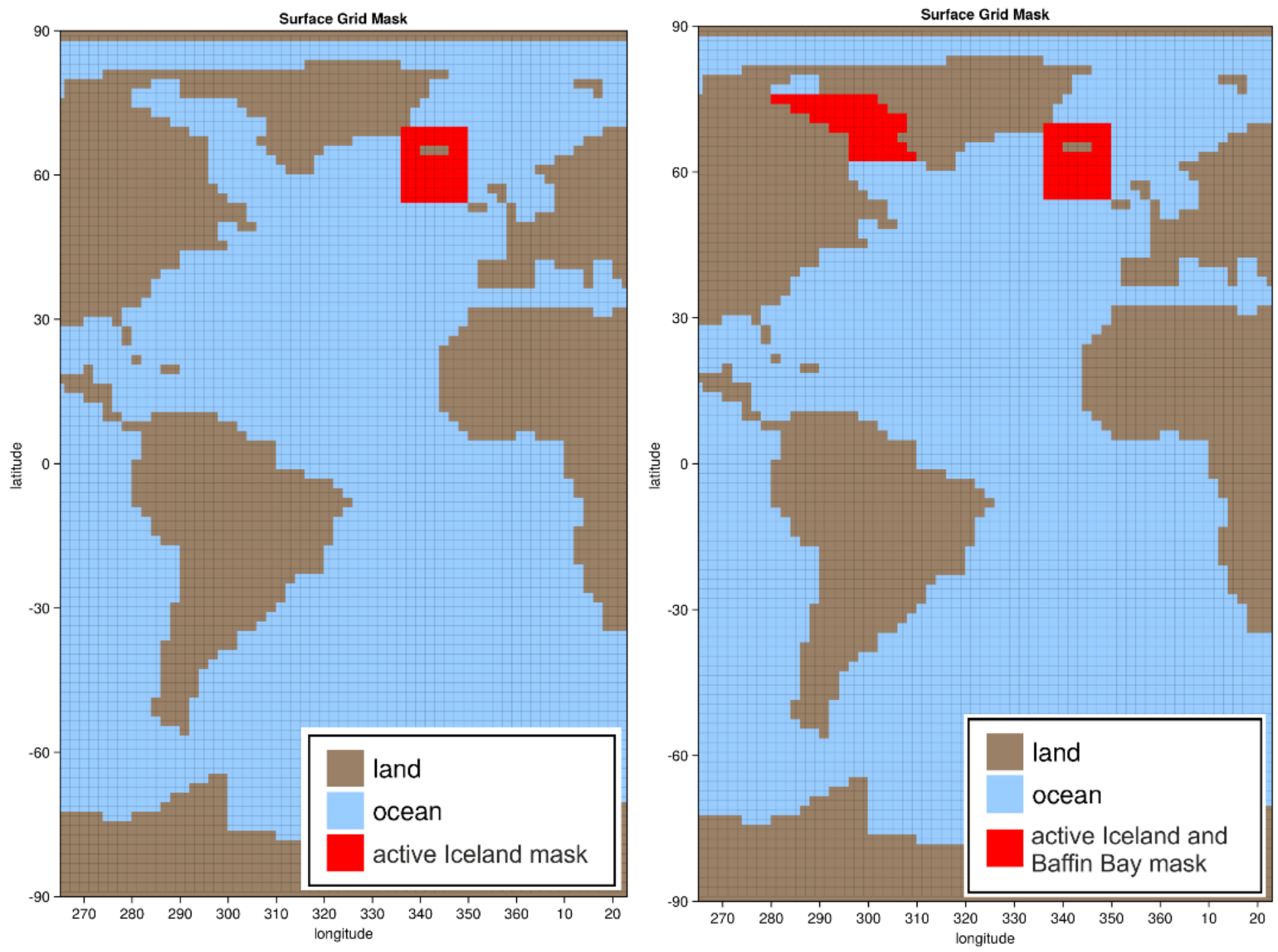

Supplementary Fig. S6. Areas defined in the NE Atlantic and Baffin Bay, assigned ε_{Nd} signatures of -7 and -28, respectively, for GNOM sensitivity experiments.

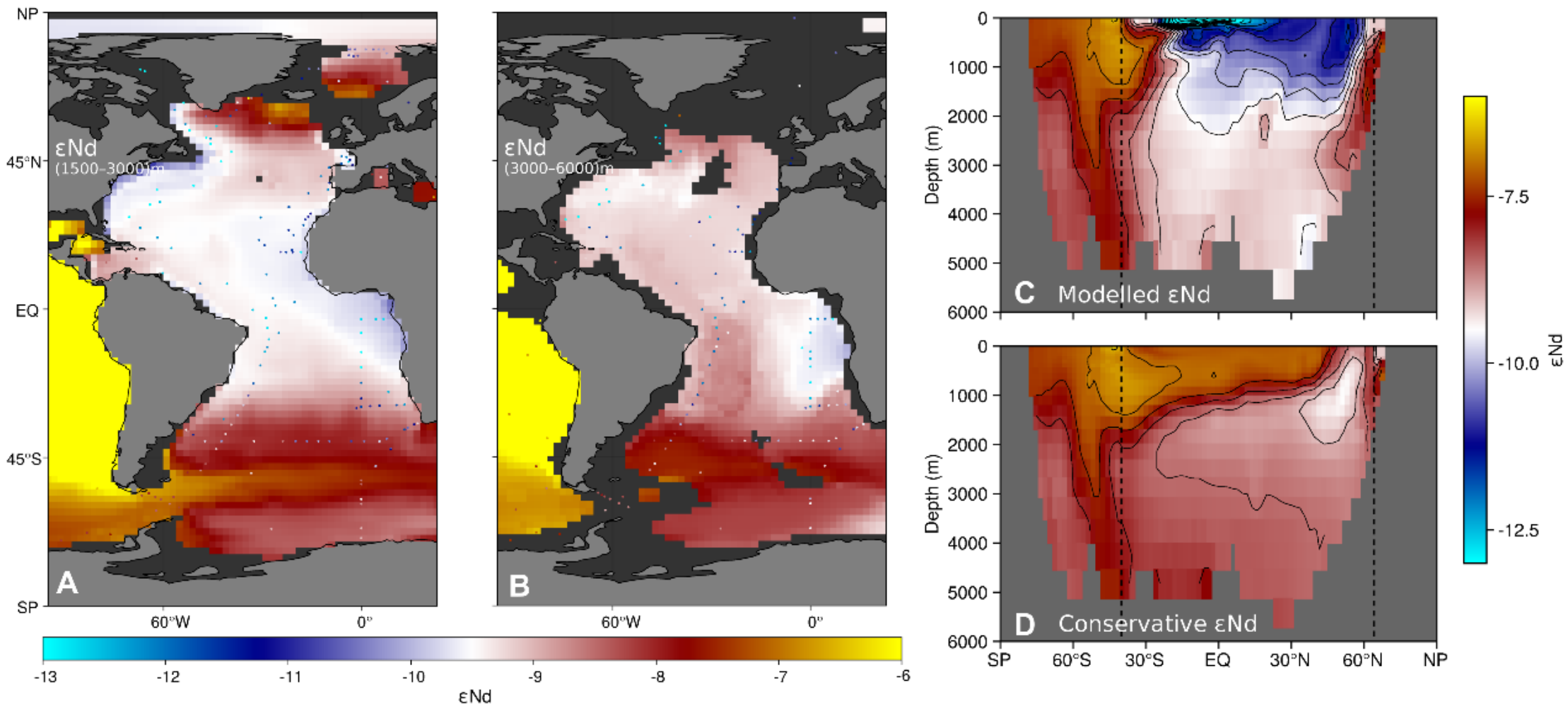

Supplementary Fig. S7. GNOM sensitivity experiment with a radiogenic eastern GNAIW endmember (ε_{Nd} = -7) prescribed in the NE Atlantic. (**A**) Modeled ε_{Nd} distribution at intermediate depths (1500–3000 m) in the Atlantic Ocean. (**B**) Modeled ε_{Nd} distribution at deep depths (3000–6000 m) in the Atlantic Ocean. (**C**) Atlantic zonal mean of modeled ε_{Nd} values. (**D**) Conservative ε_{Nd} section calculated from prescribed northern (north of 40°N) and southern (south of 30°N) Atlantic endmembers. This experiment yields mid-depth and deep Atlantic ε_{Nd} values of -9.0 to -8.5, more radiogenic than the reconstructed glacial GNAIW benchmark of approximately -10 in the mid-depth North Atlantic, indicating that an additional less radiogenic Nd source is required to balance the Icelandic contribution downstream.

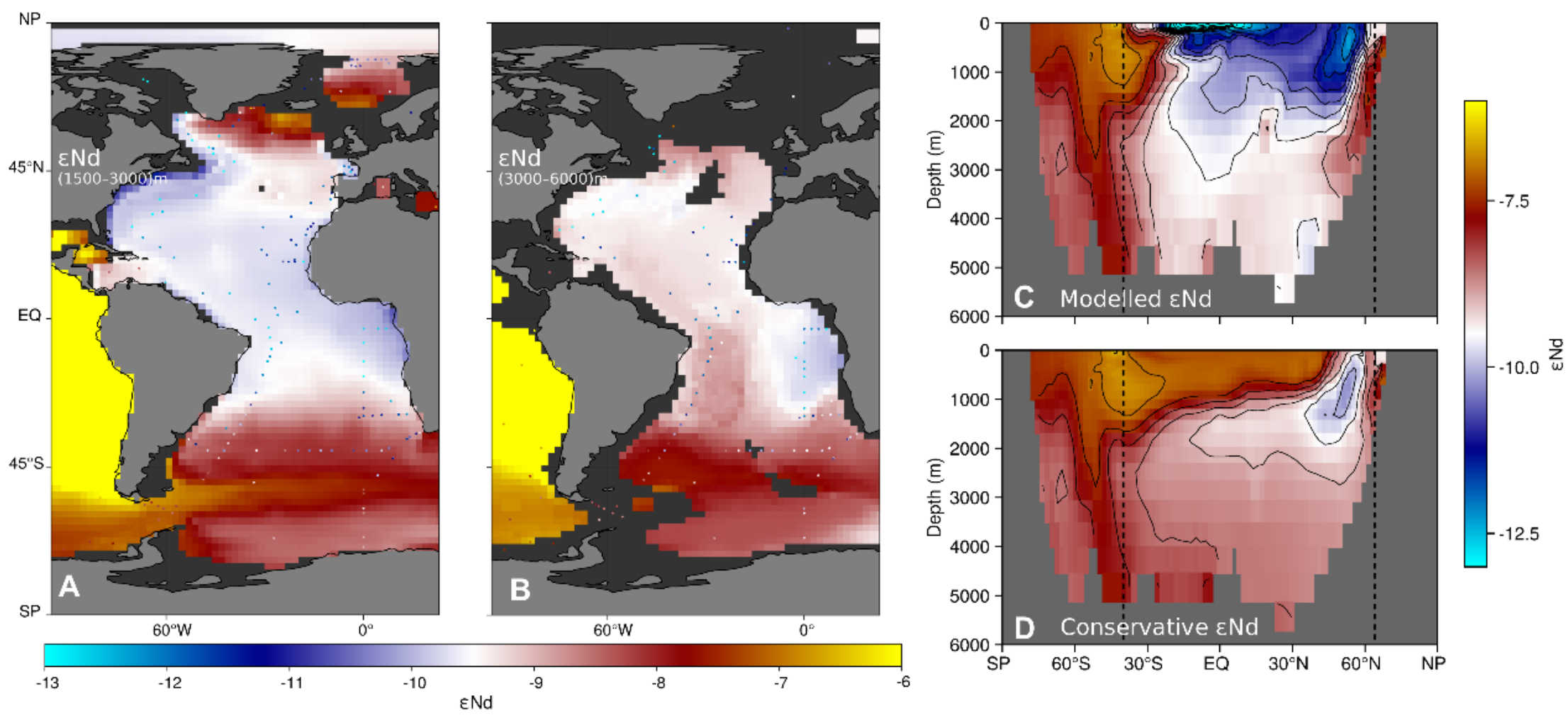

Supplementary Fig. S8. GNOM sensitivity experiment with combined radiogenic eastern and unradiogenic western GNAIW endmembers. (**A**) Modeled ε_{Nd} distribution at intermediate depths (1500–3000 m) in the Atlantic Ocean. (**B**) Modeled ε_{Nd} distribution at deep depths (3000–6000 m) in the Atlantic Ocean. (**C**) Atlantic zonal mean of modeled ε_{Nd} values. (**D**) Conservative ε_{Nd} section calculated from prescribed northern (north of 40°N) and southern (south of 30°N) Atlantic endmembers. This experiment reproduces intermediate ε_{Nd} values of approximately -10.5 to -9.5 across the mid-depth and deep Atlantic, consistent with sedimentary reconstructions, suggesting that the coexistence of a radiogenic eastern component (Icelandic inputs) and an unradiogenic western component (Laurentide and Greenland ice sheet inputs) offers a plausible explanation for the observed glacial GNAIW ε_{Nd} distribution.

5. Icelandic Nd flux reconstruction via steady-state box model

To quantify the Icelandic Nd input during glacial periods, we constructed a simple steady-state box model (Eq. 20–26, fig. S9) constrained by a mean glacial GNAIW ε_{Nd} of -7.2, an Icelandic endmember of ε_{Nd} = +6, and Atlantic inflow of ε_{Nd} = -14.5. Assuming a water-mass transport of 10 Sv, representative of glacial GNAIW volume transport, the deterministic solution yields an Icelandic Nd flux of approximately 3.9 × 10^8g yr^{-1}. To propagate endmember and transport uncertainties, we sampled 20,000 Latin hypercube realisations across plausible ranges of NE Atlantic seawater ε_{Nd} (-16.0 to -10.0), Icelandic source ε_{Nd} (+4 to +8), and effective water-mass transport (5–10 Sv) (table S4). The resulting flux distribution (fig. S10) yields a median Icelandic Nd input of 2.4 [1.0, 4.8] × 10^8g yr^{-1} (95% CI). Because radiogenic ε_{Nd} values of -8 to -4 are observed across the 1000–4000 m water column throughout much of the glacial NE Atlantic, these flux estimates are considered conservative lower bounds.

$$M_W = M_{NAC} = M_{GNAIW} \text{ (Eq. 20)}$$

$$f_{Nd^{seawater}} = M_{seawater} \times [Nd]^{initial} \text{ (Eq. 21)}$$

$$M_W \times [Nd]^{final} = M_{NAC} \times [Nd]^{initial} + F_{Nd^{Iceland}} - F_{Nd^{removal}} \text{ (Eq. 22)}$$

$$M_W \times [Nd]^{final} \times \varepsilon_{Nd^{final}} = M_{NAC} \times [Nd]^{initial} \times \varepsilon_{Nd^{initial}} + F_{Nd^{Iceland}} \times \varepsilon_{Nd^{Iceland}} - F_{Nd^{removal}} \times \varepsilon_{Nd^{final}} \text{ (Eq. 23)}$$

$$F_{Nd^{Iceland}} = \frac{M_{NAC} \times [Nd]^{initial} \times \left(\varepsilon_{Nd^{final}} - \varepsilon_{Nd^{initial}}\right)}{\varepsilon_{Nd^{Iceland}} - \varepsilon_{Nd^{final}}} \text{ (Eq. 24)}$$

$$F_{Nd^{removal}} = M_{NAC} \times [Nd]^{initial} + F_{Nd^{Iceland}} - M_W \times [Nd]^{final} \text{ (Eq. 25)}$$

$$F_{Nd^{net}} = F_{Nd^{Iceland}} - F_{Nd^{removal}} \text{ (Eq. 26)}$$

All parameter names and abbreviations have been described in the notes to Table S4.

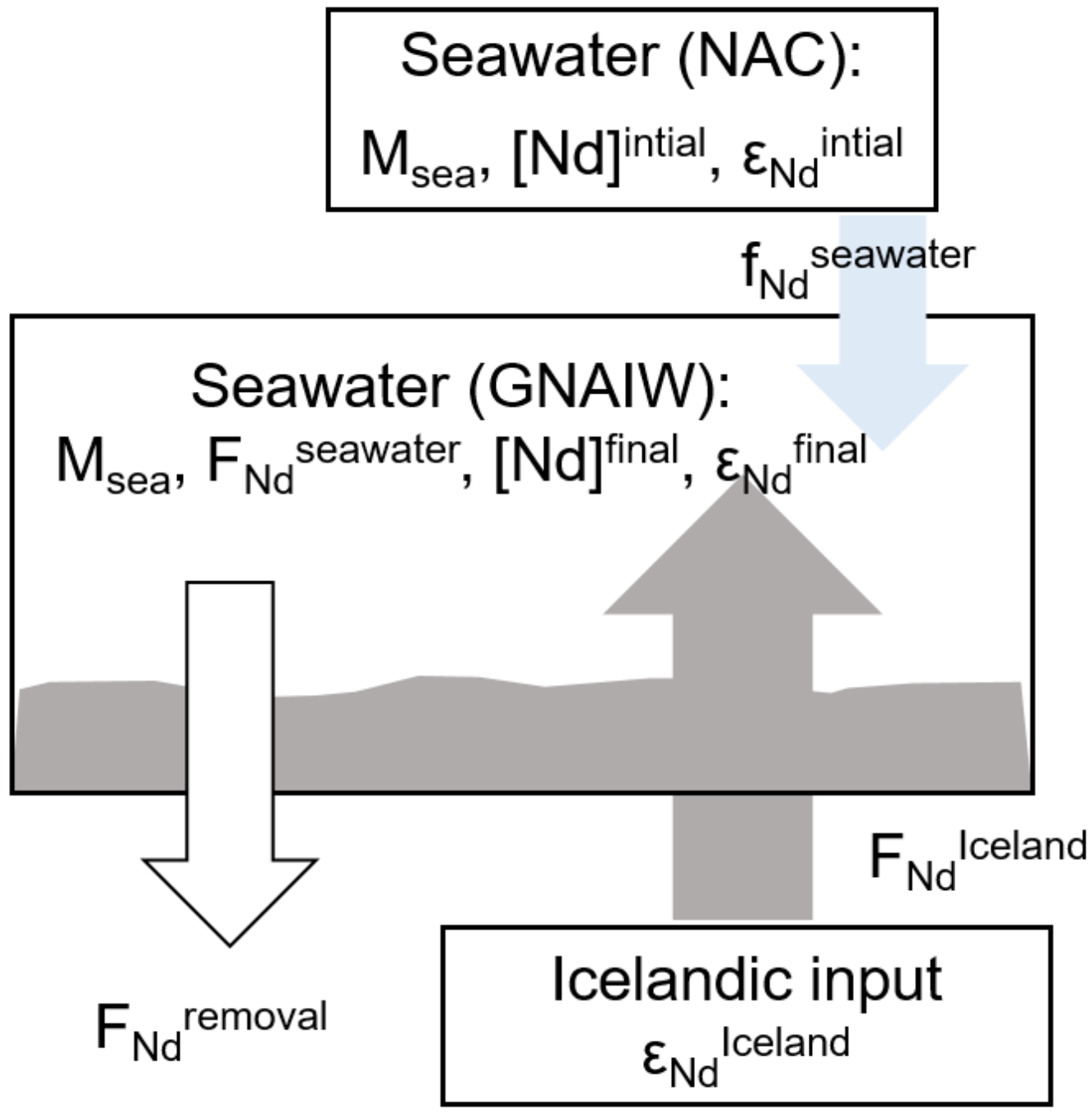

Supplementary Fig. S9. Schematic diagrams illustrating the mass balance approach used to calculate the Nd flux from the Iceland.

Supplementary Table. S4. The parameters used in the mass balance calculation.

Parameters	Values	Reference
Seawater (NAC) flux (M_{NAC}, m^3 yr^{-1})	3.2×10^{14}	(*83*)
Seawater (GNAIW) flux (M_{GNAIW}, m^3 yr^{-1})	3.2×10^{14}	(*83*)
$[Nd]^{initial}$ (pmol kg^{-1})	16.8 ± 1.3 (±1SD, n=8)	(*82*)
$[Nd]^{final}$ (pmol kg^{-1})	unknown	
$\varepsilon_{Nd}^{initial}$ (cold period)	-16.0 ~ -10.0	(*24, 12*)
ε_{Nd}^{final}	-7.2 (±1SD, n=3)	This study
$\varepsilon_{Nd}^{Iceland}$	+4 ~ +8	(*27, 49, 61*)

Note. The seawater fluxes carried by North Atlantic Current (NAC) and glacial North Atlantic Intermediate Water (GNAIW) were set to 5-10 Sv, representing a typical GNAIW (< 2000-2500m) volume transport (83). $[Nd]^{initial}$ and corresponding $\varepsilon_{Nd}^{initial}$ are the Nd concentration and ε_{Nd} of NE Atlantic seawater, respectively. ε_{Nd}^{final} represent the ε_{Nd} of GNAIW observed in this study.

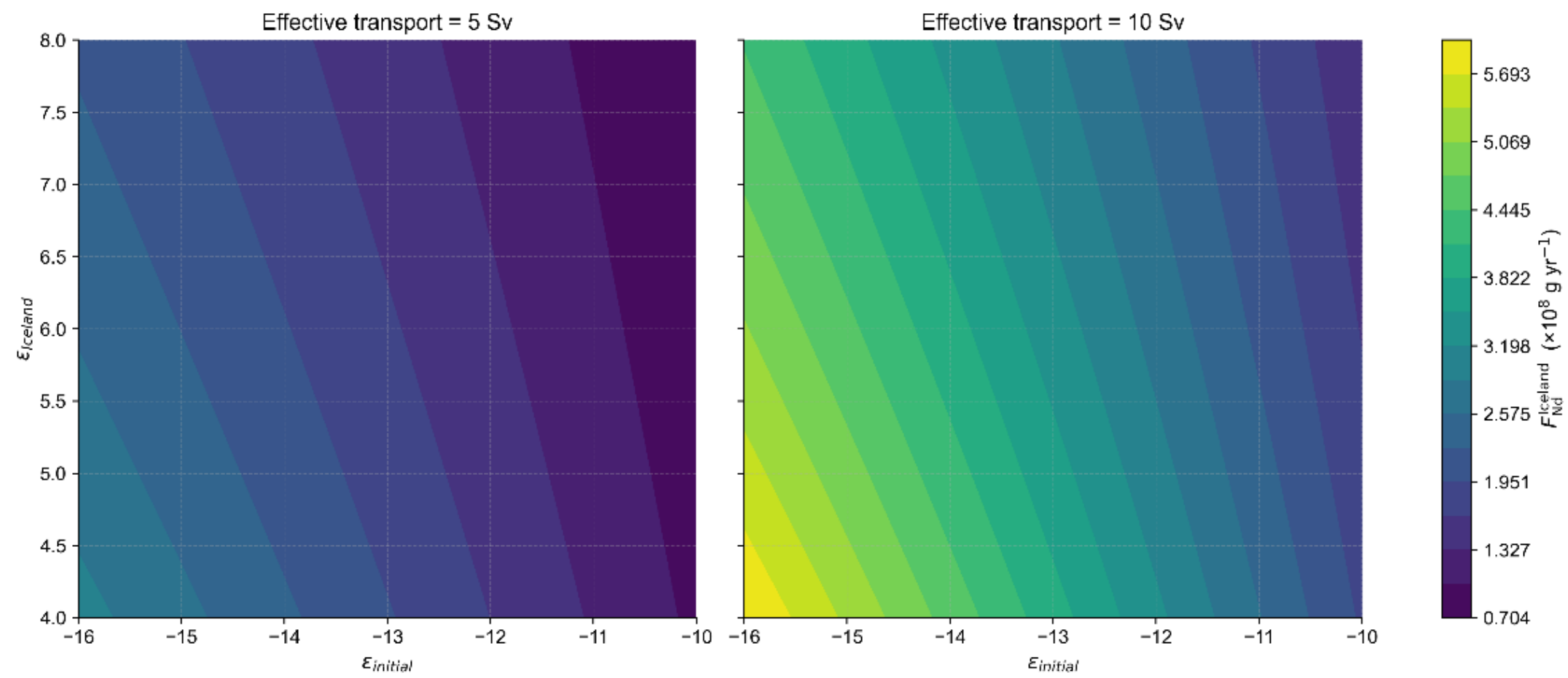

Supplementary Fig. S10. Sensitivity of the Icelandic Nd flux to endmember ε_{Nd} compositions. Contour plot showing the modeled Icelandic Nd flux ($F_{Nd}^{Iceland}$) as a function of the initial ε_{Nd} of NE Atlantic Water ($\varepsilon_{Nd}^{initial}$) and the Icelandic source ($\varepsilon_{Nd}^{Iceland}$), while the ε_{Nd} for GNAIW (ε_{Nd}^{final}) is fixed at -7.2. The calculation assumes a North Atlantic inflow of 5 and 10 Sv (left and right panels, respectively) and an initial dissolved Nd concentration of 16.8 pmol kg^{-1}. Warmer colors indicate higher Icelandic Nd fluxes required to sustain the observed ε_{Nd} value of -7.2.

Data S1. (separate file)

(1) Nd isotope compositions of Fe–Mn oxide leachates from ODP Site 982

(2) Particulate Nd isotope compositions in sediment from ODP Site 982

(3) Concentrations of major and rare earth elements of Fe–Mn oxide leachates from ODP Site 982

(4) Nd isotope compositions of secondary standard solutions and reference materials for checking the external reproducibility
